# Improving the effectiveness and equity of fuel economy regulations with sales adjustment factors

**DOI:** 10.1016/j.isci.2022.104902

**Published:** 2022-08-10

**Authors:** Shiqi Ou, Zhenhong Lin, Chieh (Ross) Wang, Stacy Davis, Shasha Jiang, Michael Hilliard, Ho-Ling Hwang, Xu Hao, Rujie Yu

**Affiliations:** 1 Buildings and Transportation Science Division, Oak Ridge National Laboratory, Knoxville, TN 37932, USA; 2 12513 Coral Reef Circle, Knoxville, TN 37922, USA; 3 School of Mechanical Engineering, University of Science and Technology Beijing, Beijing 10083, China; 4 China Automotive Technology and Research Center, Dongli District, Tianjin 300300, China

**Keywords:** Energy resources, Energy policy, Energy management, Energy modeling, Energy transportation

## Abstract

Larger vehicles, such as sports utility vehicles, consume more energy than cars. Their increasing popularity runs contrary to the goal of fuel economy regulations to reduce fossil fuel consumption and greenhouse gas emissions and can be explained by consumer preference and lower regulation stringency, which is due to footprint, truck classification, and the omission of heterogenous lifetime vehicle distance traveled among vehicle classes. This study shows that, for both the US and China, large vehicles travel more, last longer, and are owned by higher income consumers. This means large vehicles and their high-income owners use more fuel and emit more pollutants than represented by current policy and thus raises both policy effectiveness and energy equity concerns. We propose and estimate Sales Adjustment Factors that weigh fuel economy standards based on vehicle lifetime usage and demonstrate the resultant significant improvements in the effectiveness and equity of fuel economy regulations.

## Highlights

• Current fuel economy regulations ignore vehicle usage heterogeneity
• Large vehicles travel more, last longer, and are owned by higher income consumers
• Sales Adjustment Factors (SAF) are formulated and estimated as a policy improvement
• SAF impacts the industry by $0.4–$1.2 billion and consumers by up to $800/vehicle

## Graphical abstract

**Figure:**
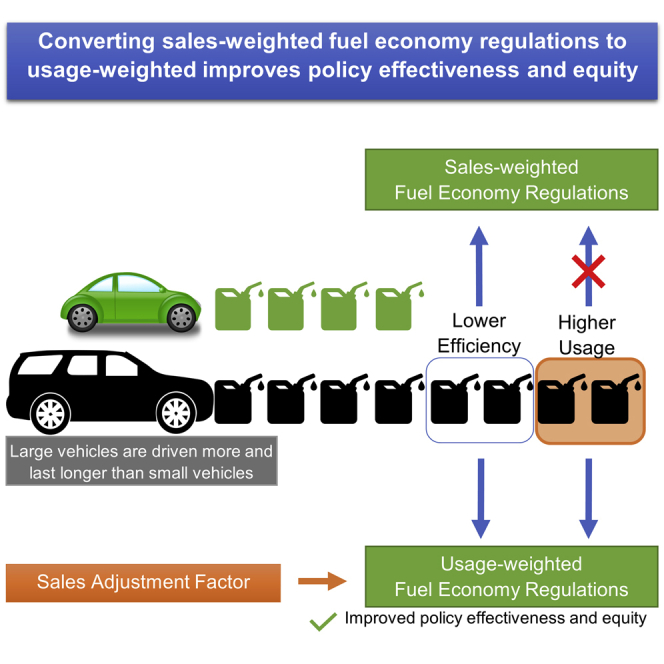

## Introduction

Tension is growing between the increasing global demand for passenger vehicles and the pressing need to reduce fossil fuel consumption and transportation CO_2_ emissions to achieve climate change goals. To promote fuel efficiency and mitigate vehicle CO_2_ emissions, sales-weighted fuel economy regulations have been adopted by almost all countries with major vehicle markets, such as the US, the European Union (EU), Japan, and China (Ou et al., 2018; NASEM 2021). The US established Corporate Average Fuel Economy (CAFE) standards in 1975 after the oil crisis (Komiyama 2008). Starting in model year 2008, the CAFE program shifted to a footprint-based approach, setting relatively less stringent fuel economy targets—in terms of miles per gallon (MPG)—for larger vehicles (NRC, 2011). Furthermore, the 2008 CAFE rulemaking created different MPG target curves for passenger cars and light trucks, further relaxing the MPG requirement for truck-based sports utility vehicles (SUVs), minivans, and pickup trucks (NHTSA 2020). Similarly, the EU regulates CO_2_ emissions per kilometer (g/km) of vehicles as the measurement for evaluating the sales-weighted fuel economy, and the EU also stipulates the linear relationship between the CO_2_ emission limits and vehicle weights (Tietge et al., 2020). China uses the fuel consumption rate (L/100 km) to evaluate the sales-weighted fuel consumption in its Corporate Average Fuel Consumption (CAFC) standard, and the fuel consumption target varies with the vehicle weight (Ou et al., 2018). Both the EU and China regulations stipulate higher (i.e., less stringent) CO_2_ emissions or fuel consumption targets for heavier vehicles. In sum, the fuel economy of an automaker is often assessed through sales-weighted or production-weighted metrics. This allows automakers some flexibility in meeting fuel economy regulations while maximizing profits with their competitive products. What is also common is that larger or heavier vehicles are required to meet less-stringent efficiency targets.

**Figure 1:**
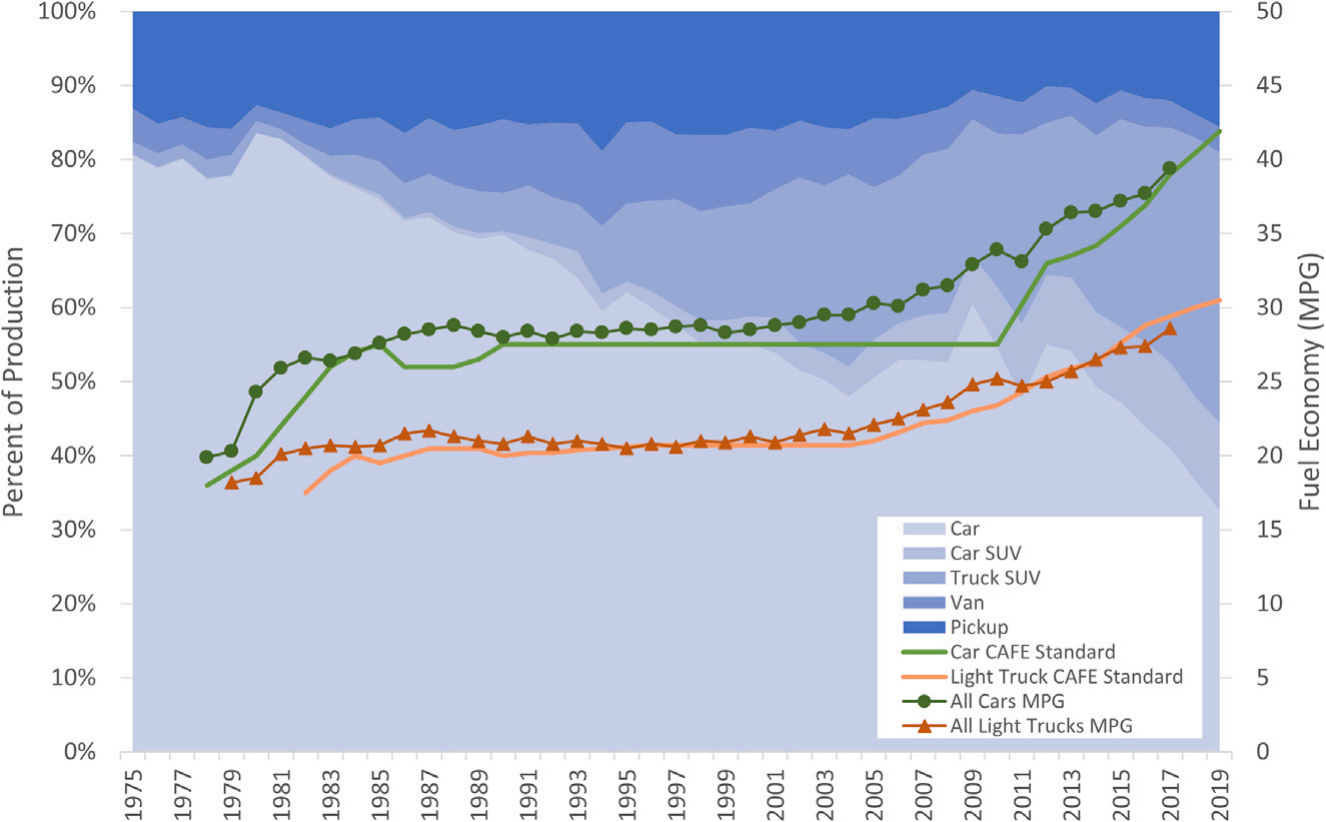
Vehicle sales shares by classes and average fuel economy over time for the US The two CAFE standard curves show required sales-weighted targets. The two MPG curves represent sales-weighted performance. Source is from the Transportation Energy Data Book (Davis and Boundy 2021).

Large vehicles (SUVs, minivans, and pickups) present a problem for fuel economy regulators. For the goal of increasing average fuel efficiency and reducing fleet-wide fuel consumption, a growing market share of small vehicles is more desirable, as they are generally more efficient. However, throughout the world, especially in the US (Figure 1) and China, larger vehicles have become increasingly popular and are known to be more profitable than smaller vehicles such as cars (Vanderheiden 2006; Snyder 2017). In the US, large vehicles accounted for 67% of the 2019 light-duty vehicle market (MarkLines 2020a). In addition, as shown in Figure 1, SUVs, especially truck-based SUVs, have gained the most market share in recent decades. The 2008 CAFE rulemaking (with the adoption of separate, footprint-based standards for cars and trucks) was followed by fuel economy performance improvements of 25% for cars and a lower 21% for light trucks by 2017, which widened the gap between car and light truck average fuel economies. In China, the sales share of cars dropped to 48% in 2019 after decreasing for several years (MarkLines 2020b). It is of policy relevance to understand what causes the conflict between the goal of fuel economy regulations and the popularity of large vehicles. Furthermore, this conflict is worsened by the fact that larger vehicles are driven more, as shown in this study.

The popularity of large vehicles can be explained by both consumer choice and supplier profit maximization (NASEM 2021). Consumers perceive better value with large vehicles with respect to capacity, comfort, safety, and functionality and are thus willing to pay more for them. From the supplier’s viewpoint, it costs more to produce larger vehicles than smaller cars, but the production cost increase is less than proportional to the increase in consumers’ willingness to pay, making large vehicles more profitable (Vanderheiden 2006; Snyder 2017). Large vehicles face less-stringent fuel economy targets under the current footprint- or weight-based regulations, which contributes to cost reduction (due to reduced burden to implement efficiency technologies) and a better value proposition, thereby discouraging the demand for small vehicles (Anderson et al., 2011; Xie and Lin 2017; Davis and Knittel 2018). Therefore, automakers that produce more small vehicles face stricter fuel economy requirements than those that produce large vehicles, which incentivizes them to upsize their products (Anderson et al., 2011; National Research Council 2015).

**Figure 2:**
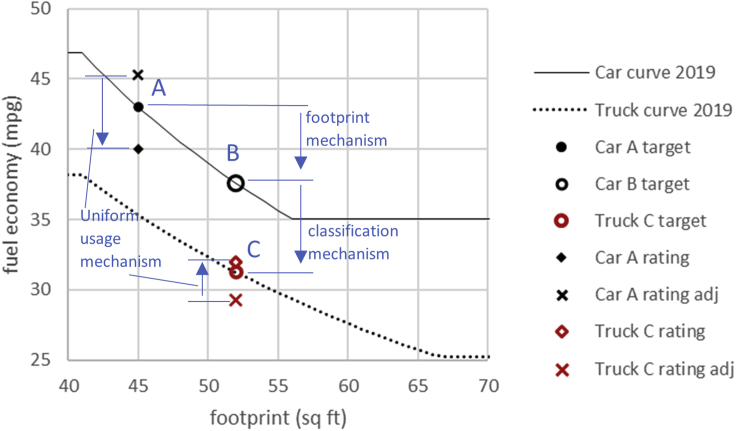
Illustration of three mechanisms leading to lower regulation stringency for large vehicles

Larger vehicles enjoy less-stringent fuel economy targets through two widely debated mechanisms: a bigger footprint (or weight) and separate classification (in the case of light-duty trucks in the US regulations) (NASEM 2021). As illustrated in Figure 2, for model year 2019, the fuel economy target is 43 MPG for Car A, a car with a footprint of 45 sq. ft. The target is relaxed by 5.4 MPG to 37.6 MPG for Car B, due to its larger footprint of 52 sq. ft. This refers to the footprint mechanism. The target is further relaxed by 6.4 MPG to 31.2 MPG if the vehicle is classified as a light truck (Truck C in the figure below has the same footprint as Car B). This relaxation of fuel economy targets is the classification mechanism. Some argue that fuel economy regulations should require small and large vehicles to adopt fuel-saving technologies and improvements equally, whereas some defend the lower-stringency targets for larger vehicles based on physical constraints of mass and aerodynamics, vehicle functionality, and consumer needs (Lutsey and Sperling 2005; Anderson et al., 2011).

The higher the fuel economy ratings of the vehicle products, the better the chance a manufacturer will meet or exceed the fuel economy standards. The fuel economy rating (“Car A rating” and “Truck C rating” on Figure 2) of a specific vehicle product is based on standard test cycles and calculation that ignores vehicle usage heterogeneity. As to be argued and explained by this study, if weighted by lifetime vehicle distance, the equivalent fuel economy ratings should be higher for small vehicles (more efficient and less driven; “Car A rating adj” on Figure 2) and lower for large vehicles (less efficient and more driven; “Truck C rating adj” on Figure 2), relative to their ratings based on test cycles. In other words, the current fuel economy ratings of large vehicles should be adjusted downward, to reflect their higher lifetime usage and greater lifetime fuel consumption. Thus, ignoring the higher usage of large vehicles reduces their regulation stringency. This effect is called the uniform usage mechanism, which is illustrated in Figure 2 and explained in “Quantifying impacts of SAF on regulation effectiveness and equity” in STAR methods. Unlike the previous two mechanisms that affect fuel economy targets, the uniform usage mechanism affects fuel economy ratings (not literally, but equivalently from a lifetime fuel consumption perspective) in favor of vehicles that are less efficient but driven more.

A factor not considered by current fuel economy regulations is that large vehicles are driven more. As reported in the literature, light trucks in the US have a higher lifetime mileage than passenger cars (Lu 2006; U.S. Environmental Protection Agency, 2016; Davis and Boundy 2021). Similarly, larger, more expensive vehicles in China are driven more (Ou et al., 2020). Vehicle usage and ownership correlate with demographic attributes (e.g., income), pointing to potential transportation energy equity concerns (Ou et al., 2020). Although the effectiveness of fuel economy regulations has been evaluated with respect to different vehicle classes/sizes (Lutsey and Sperling 2005; Anderson et al., 2011; Davis and Knittel 2018; NASEM 2021), no study has examined the effectiveness and equity of fuel economy regulations from the perspective of vehicle usage heterogeneity. Accounting for the high usage of large vehicles changes their weighted contribution in the average fuel economy calculation and thus could affect compliance strategies (Wadud et al., 2010; Lin and Greene 2011; U.S. Energy Information Administration 2021). This could lead to an increase in both the efficiency and price of large vehicles, improving both the effectiveness and equity of fuel economy regulations.

This study shows evidence that, in both the US and China, large vehicles are driven more than represented in regulations and are more commonly owned by high-income households. It also discusses the implications of these factors on the effectiveness and equity of fuel economy regulations. We then propose a simple method, called the Sales Adjustment Factor (SAF), to correct this usage underrepresentation. We formulate and estimate the SAF based on data for the US and China. With the estimated SAF, the adjusted average fuel economy (now lifetime usage weighted) is 0.41–0.56 MPG lower for the US and 0.36–0.54 MPG lower for China, depending on the model year. This translates into a significant annual compliance value of $225 million to $1,229 million for the US and $212 million to $1,047 million for China (depending on the assumed penalty rate), at least 100 times the annual compliance penalty of $2.3 million to $79.4 million paid by the US auto industry during the 2008 to 2016 model years. In terms of the equity impact, the application of SAF is equivalent to increasing fuel economy ratings for small vehicles and decreasing them for large vehicles. With SAF, the fuel economy ratings are adjusted by −4.2 MPG to +5.3 MPG for the US and by −7.5 MPG to +8.5 MPG for China, depending on vehicle class and model year. These translate into a compliance value of −$590 to +$739 per vehicle for the US and −$658 to +$750 per vehicle for China, representing a significant gain (for small vehicles) or loss (for large vehicles) per vehicle as perceived by the manufacturer. To address the additional goals of reducing petroleum consumption and greenhouse gas emissions, fuel economy regulations have evolved over time with new designs, such as attribute-based targets and special treatment for zero-emission vehicles (National Research Council 2011, 2015; NASEM 2021) (not in the study scope). This study focuses on proper lifetime usage representation and will contribute to improvements in both regulation effectiveness and energy equity.

## Results

### Current regulations ignore heterogenous vehicle usage

Fuel economy regulations are supposed to achieve energy-saving benefits throughout a vehicle’s lifetime. That is, the corporate average fuel economy should be weighted by vehicle lifetime usage. This intent is evidenced by the official cost-effectiveness analyses of fuel economy regulations that estimate fuel savings based on annual vehicle distance traveled, survival rate, and vehicle lifetime (EPANHTSA, 2010). However, these three usage-related factors are not reflected in the sales-weighted approach used in current fuel economy regulations because they are implicitly assumed to be either homogeneous or independent of fuel economy ratings, as mathematically demonstrated below. This *theoretical* finding provides a coherent framework for understanding the *empirical* findings of usage heterogeneity (“Large vehicles travel more and last longer” and “High-income households more likely to own large vehicles” in Section results) and explaining the proposed solution (“sales adjustment factors as a solution” in Section results).

Here is the mathematical reasoning. For any given model year, the originally intended usage-weighted corporate average fuel economy $CAFE_{usage}$ is a function of fuel consumption rate ($f_{i}$, amount of fuel per unit of distance), annual driving distance ($d_{i}$), vehicle lifetime ($l_{i}$), and sales $n_{i}$, for vehicle model or type i, as shown in Equation (1). The denominator in Equation (1) represents the total lifetime fuel consumption of all vehicles sold for a given regulated model year by a manufacturer, whereas the numerator represents total lifetime travel distance of those vehicles. This calculation method is called usage-weighted, as the term $n_{i}l_{i}d_{i}$ represents the lifetime usage (i.e., lifetime distance) and acts as the weighting factor, as opposed to the current fuel economy regulations that are only weighted by sales $n_{i}$. Discounting should ideally be considered, but it is ignored in this paper for simplicity.

(Equation 1) $$CAFE_{usage}=\frac{\sum_{i\in I}\left(n_{i}l_{i}d_{i}\right)}{\sum_{i\in I}\left(n_{i}l_{i}d_{i}f_{i}\right)}$$

It is obvious that annual driving distance ($d_{i}$) and vehicle lifetime ($l_{i}$) vary among vehicles. However, if the variation of either of them is independent of fuel consumption rate ($f_{i}$) and sales ($n_{i}$), or if we ignore such variation for either $d_{i}$ or $l_{i}$, we can then assume $d_{i}=d_{0}$ and $l_{i}=l_{0}$ for ∀i or we can assume E[$d_{i}l_{i}$] = $\;d_{0}l_{0}$. Based on Equation (1), we derive the sales-weighted corporate average fuel economy $CAFE_{sales}$ as in Equation (2), which represents the current sales-weighted approach used in the US, EU, and China.

(Equation 2) $$CAFE_{sales}=\frac{\sum_{i\in I}n_{i}}{\sum_{i\in I}\left(n_{i}f_{i}\right)}$$

This suggests that the current sales-weighted approach is intended to reflect the usage-weighted fuel economy and is simplified with the equal-usage or independent-usage assumption. The main point of the empirical results in the next section is to challenge this assumption and to show that both the annual driving distance ($d_{i}$) and the vehicle lifetime ($l_{i}$) in Equation (1) vary and correlate with fuel consumption rate ($f_{i}$) through their correlation with vehicle size or class. This would challenge the current sales-weighted approach. Admittedly, the fuel economy regulations are more complex than what Equations (1) and (2) describe. However, Equations (1) and (2) capture the relevant essence of fuel economy regulations that allow automakers to adjust their strategies using different vehicle classes/sizes to meet fuel economy standards while maximizing profits (Chen and Zhang 2013; Gillingham et al., 2021).

### Large vehicles travel more and last longer

Larger vehicles in general have lower fuel economies, travel more, and last longer, as shown in Table 1. For example, in the US, SUVs travel about 2% more and last about 15% longer than midsize cars. Similarly, SUVs, minivans, and crossovers in China travel more and last longer than cars, although vehicles in China travel less overall and do not last as long as those in the US. More detailed comparisons of per-vehicle annual VMT, fuel economy, and vehicle lifetime are provided in Figures S3–S6.

**Table 1:** Estimates of average fuel economy, average per-vehicle annual VMT, and expected vehicle lifetime by vehicle class/size for the US and China

Vehicle class/size	MPG	Per-vehicle annual VMT (miles)	Expected lifetime (year)
**US**			

Car			16.19
Small	26.40	9,462	
Midsize	26.01	10,434	
Large	19.96	9,553	
SUV	20.38	10,605	18.56
Minivan	19.55	11,478	
Pickup	16.18	10,449	

**China**			

Car			13.76
Small	35.91	7,510	
Midsize	30.68	9,191	
Large	29.16	10,332	
SUV/minivan/crossover			15.32
Small	30.69	9,273	
Midsize	26.15	9,982	
Large	22.36	10,838	

MPG, miles per gallon; VMT, vehicle miles traveled. Original data for calculating MPG and VMT (records-weighted) in the US are from the 2017 National Household Travel Survey (U.S. Department of Transportation Federal Highway Administration, 2017) and fueleconomy.gov (fueleconomy.gov 2020). The expected vehicle lifetime in the US is from Lu (2006) (Lu, 2006). Original data for calculating MPG and VMT in China by the end of 2018 is collected from the Chinese government and commercial websites, including the Ministry of Industry and Information Technology (https://yhgscx.miit.gov.cn), GUAZI (www.guazi.com), and BearOil (www.xiaoxiongouhao.com). The details of calculations for China’s market are presented by (Ou et al., 2020). The expected vehicle lifetime in China is from Zheng et al. (2019) (Zheng et al., 2019). Data are deposited on Mendeley Data (doi:10.17632/98mcnkssgf.1).

A large vehicle consumes more fuel annually, as shown in Table 2, which summarizes the major indicators for the annual fuel consumption per vehicle in the US and China. First, the annual fuel consumption by vehicles is positively correlated to vehicle size regardless of whether the comparison is within vehicle class (e.g., midsize cars versus large cars) or among vehicle classes (e.g., SUVs versus cars). In addition, the median values of annual fuel consumption in the US and China are smaller than the mean values, which means that the distribution of annual fuel consumption for vehicles in the US and China is right-skewed, as shown in Figures S4 and S5. The annual fuel consumption per vehicle tends to be greater in the US than in China because the annual per-vehicle VMT in the US is larger.

**Table 2:** Estimated annual fuel consumption (in gallons) by vehicle class and size

Class and size	Median	Mean	50% Distribution
**US**			

Car			
Small (two-seaters, mini-compact/subcompact/compact cars, small station wagons)	347	441	[188, 554]
Midsize (midsize cars, midsize station wagons)	388	479	[217, 603]
Large (large cars)	412	543	[219, 653]
SUV	516	625	[300, 790]
Minivan	560	663	[337, 813]
Pickup	558	714	[299, 916]

**China**			

Car/crossover			
Small (mini/compact/small cars and similar)	235	261	[171, 320]
Midsize	324	349	[241, 428]
Large (mid-large/large cars)	387	413	[273, 511]
SUV/minivan			
Small (Compact/small SUVs)	326	363	[229, 450]
Midsize (midsize SUVs, MPVs)	412	443	[300, 551]
Large (mid-large/large SUVs, minivan)	571	611	[413, 758]

Original data for calculating fuel consumption (records-weighted) in the US is from the 2017 National Household Travel Survey (U.S. Department of Transportation Federal Highway Administration, 2017) and fueleconomy.gov (fueleconomy.gov 2020). Original data for calculating MPG and VMT in China by the end of 2018 is collected from the Chinese government and commercial websites, including the Ministry of Industry and Information Technology (https://yhgscx.miit.gov.cn), GUAZI (www.guazi.com), and BearOil (www.xiaoxiongouhao.com). Data are deposited on Mendeley Data (https://doi.org/10.17632/98mcnkssgf.1).

**Figure 3:**
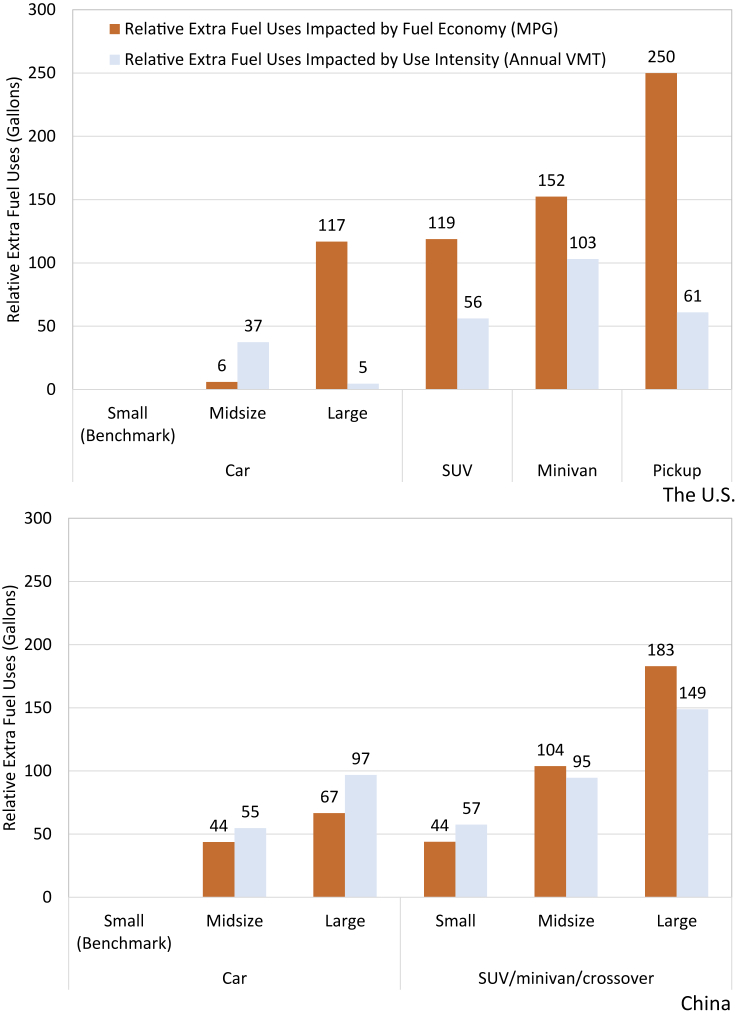
Additional fuel consumption due to vehicle fuel economy and vehicle use intensity for different vehicle types The annual fuel consumption of small cars is used as a benchmark for the US and China, respectively.

Large vehicles consume more fuel because of the combined effect of lower fuel economy and greater annual driving distance. The incremental increase in annual fuel consumption due to a lower fuel economy and a higher per-vehicle annual VMT are shown in Figure 3, using small cars in the US and China as benchmarks. The calculation of the extra fuel consumption is based on the mean values of per-vehicle annual VMTs and MPGs for different vehicle class/size, which are shown in Table 2 and Figure S3. In general, both the lower fuel economy and higher use intensity in larger vehicles cause more fuel consumption relative to the benchmarks. In the US, the extra fuel consumption caused by lower vehicle fuel economy tends to be greater than that caused by the higher vehicle use intensity for all vehicle sizes except midsize cars. This implies that fuel economy heterogeneity plays a bigger role in differentiating annual fuel consumption than the heterogeneity in use intensity does. In China, fuel economy heterogeneity and vehicle usage heterogeneity show similar contributions to variations in annual vehicle use.

Moreover, the higher annual mileage and fuel consumption rate of large vehicles are further amplified by their longer lifetime. In the US, the survival rate of light trucks with 25 years of age is higher than 12%, whereas the survival rate of passenger cars with 20 years of age is below 10% (Lu 2006). Lifetime mileage is 21% higher for light trucks than passenger cars (Lu 2006). For vehicles in China, Zheng et al. (2019) showed that the lifetime of SUVs/minivans is much longer than that of cars (Zheng et al., 2019). In theory, the longer life of large vehicles could reduce the demand for new vehicles in the future. On one hand, this reduces the GHG emission associated with vehicle production. On the other hand, this also slows down fleet turnover with efficient or electric vehicles (assuming new vehicles become more efficient or more electrified) and thus prolongs fuel consumption of on-road vehicles. However, this is uncertain and needs further research. What is clear is that the lifetime mileage and lifetime fuel consumption are much greater for larger vehicles than for smaller ones.

Usage underrepresentation of large vehicles in fuel economy regulations may contribute to their popularity. It reduces the efficiency improvement pressure on larger vehicles, which is in addition to the relaxation effect from less-stringent fuel economy targets. The lower pressure to improve efficiency may contribute to the lower manufacturing cost, the market popularity, and the high profitability of large vehicles, incentivizing automakers to make and sell more of them. This works against the goal and effectiveness of fuel economy regulations.

### High-income households more likely to own large vehicles

Households show heterogeneity in driving intensity and fuel consumption corresponding to vehicle class and size (Department of Transportation. 2017). Vehicle purchase preferences and vehicle use characteristics could vary among households, and vehicle fuel consumption could also vary with household characteristics, such as income, household size and composition, and location. The Lorenz Curve is adopted to graphically represent the inequality in fuel consumption, as shown in Figure S7. In 2017, the top 28.3% of US households consumed 50% of the fuel. In addition, all US households are further divided into four groups based on cumulative fuel consumption percentiles. With all households sorted in the order of their fuel consumption from low to high, Groups 1, 2, 3, and 4 consist of the households that make up the first, second, third, and fourth quartiles of cumulative fuel consumption. Group 1 (lowest per-household fuel consumption) and 4 (highest) consist of about 46.5% and 10.3% of US households, respectively.

**Figure 4:**
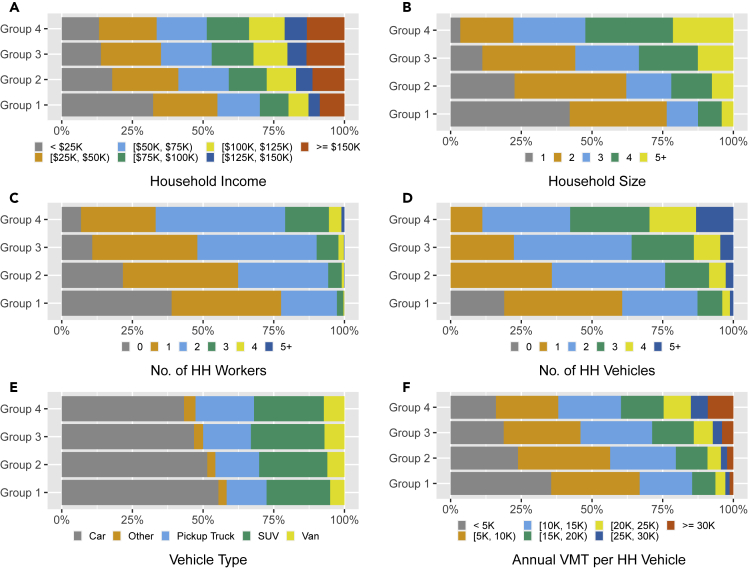
Comparison of household and vehicle characteristics Groups 1, 2, 3, and 4 consist of the households that make up the first, second, third, and fourth quartiles of cumulative fuel consumption in the US. The original data are from the National Household Travel Survey (U.S. Department of Transportation Federal Highway Administration, 2017). (A) Household income by fuel consumption group; (B) Household size by fuel consumption group; (C) Numbers of household (HH) workers by fuel consumption group; (D) Numbers of household vehicles by fuel consumption group; (E) Vehicle types by fuel consumption group; and (F) Annual VMT per household vehicle by fuel consumption group.

In Figure 4, some major demographic characteristics of the four groups are compared to explore potential underlying characteristics that might contribute to the observed heterogeneity in vehicle usage. As observed in Figures 4A–4D, when compared with Groups 1 and 2, Groups 3 and 4 show higher percentages of households with higher income, larger household size, more workers, and more vehicles. This result—higher-income and larger households are likely to consume more fuel—intuitively makes sense. What’s more, households from these groups show differences in the types of vehicles they own and how many miles they drive annually, as shown in Figures 4E and 4F. The households from Group 4 are not only more likely to own larger vehicles (e.g., SUVs, pickup trucks, and vans) that typically have lower fuel economies, but they also tend to drive these vehicles more. A complete econometric analysis of the endogeneity between the household incomes and fuel consumption (affected by vehicle types and per-vehicle annual VMT) is provided in “Quantifying the relationship between household incomes and per-vehicle annual fuel consumptions” in STAR methods and Figure S8. The analysis shows that the households with higher incomes consume more fuel, which correlates with vehicle size and vehicle driving intensity. This conspicuous imbalance of fuel consumption among households with different incomes is also found by other studies (Greene and Welch 2018). In summary, households with higher incomes are more likely to own larger vehicles, drive more, and therefore consume more fuel.

Unlike the US, China has no comprehensive survey of data regarding household travel. Therefore, it is hard to quantify the relationship between household characteristics and vehicle fuel consumption. However, we can still infer some relationships through available literature. Ou et al. found that per-vehicle annual VMT is positively correlated with vehicle price and vehicle class/size (Ou et al., 2020). A higher household income often implies a higher probability of being able to afford a more expensive vehicle. Zheng and Huo (2010) found that people with higher incomes in Beijing are more likely to drive vehicles and that CO_2_ emissions by vehicles from households with high incomes are much greater than average (Zheng and Huo 2010). Therefore, vehicle fuel consumption could be positively correlated to household income in China as well.

Ignoring the heterogeneity of vehicle usage by vehicle class/size or, more specifically, underrepresenting usage of large vehicles in fuel economy regulations could raise energy equity concerns. Large vehicles would face more pressure for efficiency improvement if their higher usage were properly represented in fuel economy regulations. This would likely result in large vehicles that were more efficient although more expensive. On one hand, lower-income households might benefit more from improving fuel economy when considering fuel cost savings, as fuel costs are a relatively high proportion of their income (Greene and Welch 2018). Therefore, policy designs that discourage efficiency improvement could affect low-income consumers and should be examined for energy equity impacts. Furthermore, as we found, larger vehicles are more likely purchased by higher income households who also consume more fuel. This means that because of the usage underrepresentation of large vehicles in fuel economy regulations, high-income vehicle owners are more likely than low-income consumers to avoid the responsibility of paying for the efficiency improvements for large vehicles.

### Sales adjustment factors as a solution

As found thus far, large vehicles consume more fuel for three reasons: lower fuel economy (i.e., higher fuel consumption rate), a higher per-vehicle annual VMT, and a longer vehicle lifetime. The fuel consumption rate factor has been reflected in the current sales-weighted CAFE formula, whereas the other two factors have been ignored. We propose a simple modification to the current sales-weighted CAFE formula, using a Sales Adjustment Factor (SAF) as a vehicle sales multiplier (as derived by “Calculation of sales adjustment factors by vehicle class/size” in STAR methods and shown in Table 3), so that the CAFE formula can reflect the actual heterogenous lifetime usage among vehicles.

**Table 3:** Estimates of sales adjustment factors by vehicle class for the US and China

Vehicle class/size	MPG	Per-vehicle annual VMT (miles)	Expected lifetime (years)	SAF		
				Method #1	Method #2	
					Mean	90% confidence interval of mean
**US**						

Car						
Small	26.40	9,462	16.19	1.000	1.001	±0.00499
Midsize	26.01	10,434		1.103	1.146	±0.00591
Large	19.96	9,553		1.010	1.243	±0.00917
SUV	20.38	10,605	18.56	1.285	1.283	±0.00732
Minivan	19.55	11,478		1.390	1.880	±0.02280
Pickup	16.18	10,449		1.266	1.319	±0.00842

**China**						

Car						
Small	35.91	7,510	13.76	1.000	1.000	±0.00408
Midsize	30.68	9,191		1.224	1.196	±0.00446
Large	29.16	10,332		1.376	1.383	±0.00581
SUV/minivan/crossover						
Small	30.69	9,273	15.32	1.374	1.341	±0.00544
Midsize	26.15	9,982		1.479	1.424	±0.00546
Large	22.36	10,838		1.606	1.579	±0.00649

SAF, sales adjustment factor. SAF calculation uses Small Car as the reference for the US and China, respectively. Original data for calculating fuel consumption (records-weighted) in the US are from the 2017 National Household Travel Survey (US Department of Transportation Federal Highway Administration, 2017) and fueleconomy.gov (fueleconomy.gov 2020). Original data for calculating MPG and VMT in China by the end of 2018 is collected from the Chinese government and commercial websites, including the Ministry of Industry and Information Technology (https://yhgscx.miit.gov.cn), GUAZI (www.guazi.com), and BearOil (www.xiaoxiongouhao.com). Data are deposited in Mendeley Data (https://doi.org/10.17632/98mcnkssgf.1).

Small cars are used as the reference for the US and China (the result will not be affected if a different vehicle class is chosen as the reference because the usage weights are all relative). Based on method #2 (discussed by “Calculation of sales adjustment factors by vehicle class/size” in STAR methods), the SAF estimates for the US suggest that each unit of SUVs sold should be counted as 1.283 units in the CAFE regulation to reflect an SUV’s high usage. For China, the usage heterogeneity is greater. The results suggest that a unit sale of large SUVs should be counted as 1.579 units in the fuel economy regulation. Note that the SAF adjustment is intended to capture the lifetime usage heterogeneity and only affects the average fuel economy *performance*. It does not affect the determination of the footprint- or weight-based fuel economy *targets* or the standard separation for passenger cars and light-duty trucks in the US. Whether or not these footprint-based MPG targets should be adjusted is outside the scope of this paper. The SAF adjustment corrects the large-vehicle usage underrepresentation and transforms the current sales-weighted fuel economy regulations into usage-weighted regulations.

The SAF estimates in Table 3 are based on vehicle class and are implemented by multiplying them by the sales of the corresponding vehicle class. It may be necessary to further study the correlation of SAFs with other vehicle attributes, such as engine displacement and vehicle fuel type. The ultimate goal is to discover the correlation between the lifetime usage and fuel-economy-relevant attributes (such as vehicle class) or, ideally, the direct correlation between lifetime usage and fuel economy rating. If data are available and the correlation is found, an SAF with greater granularity can be estimated.

**Figure 5:**
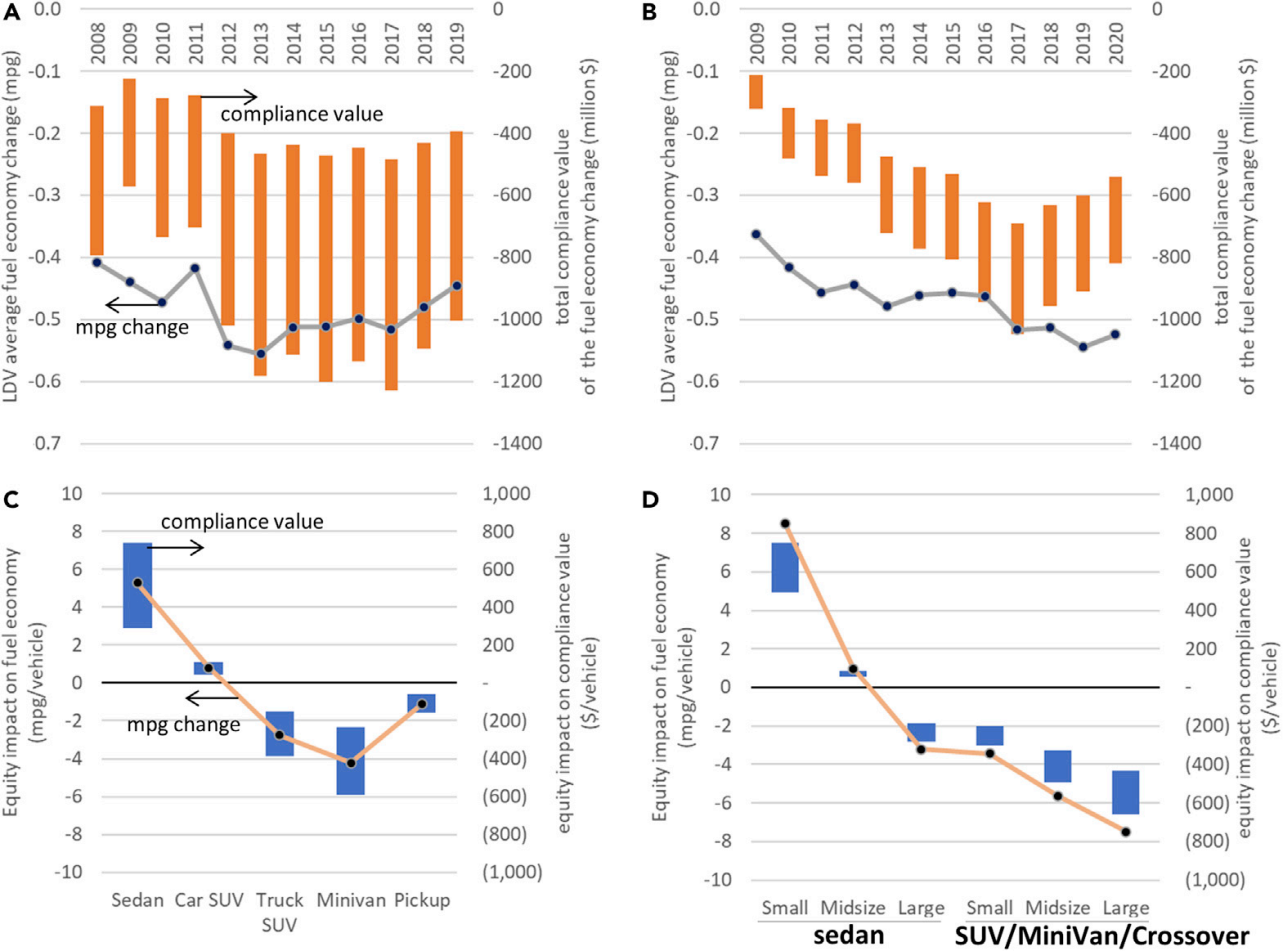
SAF impacts on effectiveness and equity of fuel economy regulations (A) Change in US light-duty vehicle average fuel economy (line) and the resulting change in total compliance value (bar) due to SAF; for compliance value bars, lowest magnitude estimate is based on the current penalty rate of $5.5 per 0.1 MPG, and highest magnitude estimate is based on the rule-making-proposed $14 per 0.1 MPG. (B) For China, lowest magnitude estimate based on $5.8 per 0.1 MPG and $8.8 per 0.1 MPG according to compliance cost estimates for 2030 and 2025 fuel consumption regulation scenarios by Wang et al. (2019). (C) Equity impact of SAF on equivalent fuel economy (line) and on vehicle-level compliance value (bar); for 2019 new vehicles in the United States; lowest and highest magnitude estimates for the bars are based the same $5.5 and $14 per 0.1 MPG as in (A). (D) For 2020 new vehicles in China; lowest and highest magnitude estimates for the bars are based on the same $5.8 and $8.8 per 0.1 MPG as in (B).

Although SAF does not address the issue of less stringent MPG targets for large vehicles, it reduces the average sales-weighted fuel economy, makes compliance more difficult, motivates vehicle efficiency improvements (especially for large vehicles), and can ultimately increase fleet-wide average fuel economy. The SAF thus enhances the effectiveness of fuel economy regulations. As shown in Figure 5, the SAF values from Table 3 result in a decrease in LDV sales-weighted average fuel economy by about 0.41–0.56 MPG during the 2008–2019 model years for the US and by about 0.36–0.54 MPG during the 2009–2020 model years for China. These can be interpreted as the upward bias of LDV average fuel economy performance due to ignoring vehicle usage heterogeneity. The magnitude of this bias, or the correction effect of the SAF, is significant, as the above MPG reductions translate into $225 million to $1,229 million of annual compliance value for the US and $212 million to $1,047 million per year of compliance value for the Chinese auto industry (Figures 5A and 5B), depending on model year and assumption of a penalty rate between $5.5 and $14 for the US (based on current and proposed penalty rates [NASEM 2021]) or between $5.8 and $8.8 for China (based on estimates of compliance cost for 2030 and 2025 [Wang et al., 2019]) per 0.1 MPG per vehicle. For comparison, during 2008–2016, the total annual compliance penalty paid by the US auto industry ranged from $2.3 million to $79.4 million, one to two orders of magnitude lower than the compliance value impact of SAF. This suggests that the significant loss in compliance value due to SAF may motivate the auto industry to adopt more efficient technologies to raise the fuel economy performance of large vehicles. It may increase the prices of large vehicles and stimulate sales of small vehicles to some extent.

In addition to the effectiveness impact, SAF could also have an equity impact, which can be measured by equivalent changes in fuel economy and vehicle-level compliance value among vehicle classes. SAF corrects the usage underrepresentation of large vehicles by increasing their lifetime fuel consumption representation in the regulations. The increase in represented lifetime fuel consumption is factually based on a higher lifetime usage, but it can be mathematically viewed as resulting from a decrease in fuel economy performance under the assumed homogeneous average lifetime usage. Similarly, SAF corrects the usage overrepresentation of small vehicles by mathematically increasing their fuel economy performance. In other words, SAF compensates small vehicles and penalizes large vehicles in terms of their fuel economy ratings, mathematically reflecting the usage heterogeneity and correcting the representation of lifetime fuel consumption in regulations. This equivalent change in fuel economy ratings allows measurements of equity (i.e., gain and loss) among vehicle classes.

The SAF can improve the social equity of fuel economy regulations indirectly by improving the “equity” among vehicle classes, given the demonstrated association between vehicle size classes and income of car owners in Section limitations of the study. The SAF values from Table 3 result in upward MPG adjustments (gains) for smaller vehicles and downward MPG adjustments (losses) for larger vehicles (Figures 5C and 5D). The adjustments vary among vehicle sizes from −4.2 MPG to +5.3 MPG for the US and from −7.5 MPG to +8.5 MPG for China. Assuming the aforementioned penalty rates for the US and compliance cost for China, the MPG adjustments result in a compliance value of −$590 to +$739 per vehicle for the US and −$658 to +$750 per vehicle for China. These represent a significant amount of gain or loss per vehicle for the manufacturers. As shown in Figures 5C and 5D, for both the US and China, SAF results, on average, in gains for small vehicles and losses for large vehicles. These gains and losses are measured in changes in compliance value for each sold vehicle as perceived by manufacturers under the SAF-adjusted fuel economy regulations. Logically, the gain for small vehicles could motivate manufacturers to reduce the price of those vehicles in order to sell more of them than they would without SAF. The loss in compliance value for large vehicles could motivate manufacturers to adopt efficiency technologies for them, as the less-efficient large vehicles are now given more weight in the corporate average fuel economy calculation. The adoption of efficiency technologies in large vehicles will theoretically drive up their price. This will have two effects: (1) improving the relative market competitiveness of small (and more efficient) vehicles and partially addressing the large-vehicle popularity issue and (2) having high-income consumers pay more for large vehicles, justified by proper usage representation in fuel economy regulations, and thus partially addressing the equity issue of large-vehicle usage underrepresentation in fuel economy regulations.

## Discussions

Contrary to the policy goals of reducing petroleum consumption and CO_2_ emissions, the increasing popularity of large vehicles is not only driven by consumer preferences but can also be attributed to the less stringent fuel economy regulations for these vehicles. This includes the well-discussed lower per-vehicle fuel economy targets due to their larger footprint, greater weight, or truck classification eligibility, as well as the possible usage underrepresentation of large vehicles (associated with lower fuel economy ratings) in fuel economy regulations, which has rarely been discussed and is the focus of the study.

This study showed evidence of the heterogeneity in both annual driving distance and vehicle lifetime among vehicle classes and of the correlation between large vehicle ownership and household income. Specifically, this study examined the hypothesis that large vehicles are driven more and are more likely owned by high-income households, and it discussed the implications of and solutions for improving the effectiveness and equity of fuel economy regulations. Using 96,979 vehicle records from the National Household Travel Survey (NHTS) for the US market and more than 50,000 vehicle records from public websites for the Chinese market, this study made several observations discussed below.

First, based on mathematical inference, the current sales-weighted fuel economy formula is, with the implicit assumption of homogenous or independent lifetime distance among fuel economy levels, a simplified version of the originally intended usage-weighted fuel economy formula.

Second, large vehicles are driven more and last longer in both the US and China, resulting in a higher vehicle lifetime usage. This means that the higher usage of large vehicles is underrepresented in the current sales-weighted fuel economy regulations for both the US and China. Usage underrepresentation of large vehicles may reduce the pressure to implement efficiency technologies and thus may contribute to the market popularity and high profitability of these vehicles. In other words, usage underrepresentation of large vehicles weakens the effectiveness of fuel economy regulations in reducing petroleum consumption and CO_2_ emissions.

Third, ownership of large vehicles is correlated with high-income consumers. This, together with large vehicles’ lower fuel economy ratings and usage underrepresentation, means that high-income consumers consume more vehicle fuel than represented in fuel economy regulations, even though they pay more on total fuel cost. High-income consumers own more large vehicles and avoid paying for efficient technologies that would have been implemented on large vehicles if their high usage were properly represented in fuel economy regulations. Therefore, usage underrepresentation of large, less-efficient vehicles also weakens the equity of fuel economy regulations.

Based on these findings and implications, we recommend that heterogeneity of vehicle usage be reflected in fuel economy regulations when vehicle usage is correlated with fuel economy ratings. Specifically, based on this study, the high usage of large vehicles should be represented in fuel economy regulations. This study formulates and proposes the SAF approach as a solution framework and provides our initial SAF estimates. The SAF adjustment can significantly improve regulation effectiveness by creating additional efficiency improvement pressure, quantified to be a 0.36 to 0.56 MPG reduction in fleet average fuel economy or $212 million to $1,229 million in annual compliance value. The adjustment can also significantly improve regulation equity by rewarding small vehicles, which are more efficient, driven less, and owned by lower income consumers, with up to an 8.5 MPG increase or $750 compliance value gained per vehicle, and by penalizing large vehicles, which are less efficient, driven more, and owned by higher income consumers, with up to a 7.5 MPG reduction or $658 compliance value lost per vehicle. We recommend further consensus studies to re-estimate SAFs with more comprehensive and granular data (e.g., for possible correlation between usage and more detailed vehicle types, brands, or even socio-demographic groups), if found necessary. SAF estimates may evolve over time due to changes in travel patterns and vehicle durability and should be updated periodically. The potential implications of the SAF adjustment on the labor market and social welfare should also be studied as alternative perspectives to the compliance value discussion in this study.

## Limitations of the study

This study has several limitations and caveats that need further investigation in the future. First of all, this article implicitly assumes that the people at a higher income level tend to own more expensive vehicles. This assumption is reasonable but still needs quantitative validations. Secondly, because of the vehicle classification limitations, the SAF values obtained from this article are for general market analysis and might not be directly used for a specific market or automaker. In addition, this study focuses on the fuel economy standards for conventional vehicles only and does not extend the analysis to the electric vehicles, which have been rapidly developing in the market. We recommend future research to collect data of electric vehicle usage and lifetime and analyze their fuel economy implications.

## Supporting citations

The references (CATARC 2020), (Department of Transportation. 2017), (Meyer et al., 2003), (NHTS 2017), and (Ou et al., 2020) appear in the supplementary information.

## STAR★Methods

### Key resources table

**Table:** 

REAGENT or RESOURCE	SOURCE	IDENTIFIER
**Deposited data**		

China vehicle market and technology information	Guizi Website	www.guazi.com
China vehicle fuel consumption rates	Xiaoxiong Youhao	www.xiaoxiongouhao.com
China vehicle class and vehicle features	Auto Home	www.autohome.com.cn
U.S. vehicle class and vehicle features	U.S. Fuel Economy Website	https://fueleconomy.gov
U.S. household travel patterns and vehicle usage data	National Household Travel Survey	www.nhts.ornl.gov

**Software and algorithms**		

Excel®	Microsoft	www.microsoft.com
@Risk	Palisade	https://www.palisade.com/risk

### Resource availability

#### Lead contact

Further information and requests for resources and method details should be directed to and will be fulfilled by the lead contact, Zhenhong Lin (linz@ornl.gov).

#### Materials availability

This study did not generate new unique reagents.

### Method details

#### Data collection and analysis

To examine how driving distance and vehicle lifetime correlate with fuel consumption rate and vehicle size, vehicle records and related model information regarding the U.S. market are collected primarily from the NHTS 2017 data set (nhts.ornl.gov) and FuelEconomy.gov (the official U.S. government source for fuel economy information). Additional information from some vehicle sales platforms (such as www.edmunds.com) is used for correction and verification. For the Chinese market, this study collected vehicle information from commercial and government websites, including GUAZI (www.guazi.com) for the VMT and vehicle feature information, and the Ministry of Industry and Information Technology (https://yhgscx.miit.gov.cn) and BearOil (www.xiaoxiongouhao.com) for the fuel economy information. Processes on data collection are described by Figure S1. Features of vehicle use intensity by class/size are described by Figures S2 and S3, and Table S1. In the U.S., the probabilities of the per-vehicle annual fuel consumption of each vehicle type/class are quantified using the information supplied by “final household weight” in NHTS and household shares by state. In China, the probabilities of the per-vehicle annual fuel consumption of each vehicle type/class are estimated through uncertainty quantification using @Risk®, an add-on to Microsoft Excel that allows the use of Monte Carlo simulation. More detailed calculation methods on per-vehicle annual VMT distributions are given by the following section - Data analysis on the vehicle records. More information can refer to Figures S4 and S5, and Table S2.

#### Collecting and integrating the vehicle and travel data in the U.S.

The NHTS provides information on individual and household travel behavior, along with characteristics of the people traveling, their household, and their vehicle makes and models. The 2017 NHTS data are used for demographic characteristics and travel analysis. Records for 256,166 vehicles (including light-duty vehicles, recreational vehicles, motorcycles, etc.) owned by households in the U.S. were collected in the NHTS. 96,978 light-duty vehicle records are used for analysis in this study. Vehicle information includes (a) make, model year, and some specific trim levels, (b) vehicle age, varying from 1 to 40 + years, (c) vehicle mileage recorded by odometer and BESTMILE (the best estimate of annual vehicle mileage by NHTS analysts) (NHTS 2017), and (d) locations of surveyed households at the city level in all U.S. states.

The NHTS provides much vehicle information such as fuel economy, make and model. Combining the NHTS classification and FuelEconomy.gov data, this study derives more detailed size classification (small, midsize, and large etc.) for each make and model, which are critical for quantifying the correlations of vehicle driving intensity (per-vehicle annual VMT) and fuel economy with vehicle class/size and household features in this study. As shown in Figure S1, the information on vehicle fuel economy by vehicle size, model, and year, including labeled fuel economy (tested by the driving cycles from the U.S. Environmental Protection Agency, or EPA) for 860 different vehicle models is obtained from FuelEconomy.gov and verified with commercial vehicles sales platforms. Because some vehicle model information covered in NHTS is incomplete, the matching between NHTS data and information from the fuel economy website could result in some records being filtered out of the analysis.

#### Collecting and integrating the vehicle and travel data in China

The Chinese vehicle market is unlike the U.S. market where we can find official surveys or databases on household travel behavior and vehicle information. To investigate the correlations between driving intensity and vehicle class/size, this study scraped data records on vehicle information (including information on vehicle model, year, price, technical specifications, and odometer mileage) from the GUAZI website (www.guazi.com), a commercial website that offers a trading platform for used cars. In total, information on 169,292 used privately-owned vehicle records was collected from the GUAZI website for VMT analysis. 57,237 vehicle records are used for VMT-fuel economy analysis in this study. No commercial or government-owned passenger vehicles are included. Based on analyses by (Ou et al., 2020), features of these records have demonstrated that they are well representative of the 2019 Chinese passenger vehicle market. The information for each vehicle record includes (a) brand, model year, and specific trim level, for example: MINI 2014 1.5T COOPER Fun; (b) vehicle age measured by months, varying from 2 to 180 months; (c) vehicle mileage recorded by odometer, which ranged from 100 to 300,000 km; (d) vehicle price, specifically manufacturer’s suggested retail price (MSRP), varying from 24,000 to 1,048,800 CNY (or $3,473 to $ 151,780); and (e) location of vehicle registration at the city level. Records were collected for 26 cities across 9 provinces in China. In addition, through data mining, this study also obtains information on labeled fuel economy, vehicle size, and price range by vehicle brand, model year, and specific trim level from BearOil (www.xiaoxiongouhao.com), a smartphone application that helps users record their historical fuel costs and calculate self-reported MPG after refueling (Ben Dror et al., 2019). The vehicle’s labeled fuel economy is certified by China’s Ministry of Industry and Information Technology (MIIT) after testing with the New European Driving Cycle.

As shown in Figure S1, vehicle fuel economy information for specified brands, models, and trim levels from BearOil is matched with used car data collected from GUAZI. Due to the differences in the nomenclatures of vehicle size and classification between these two data sets, we defined a data matching method for integrating the data sets. In order of their priority, these logic judgements of the proposed method are described below:

• Accurately match the manufacturer and the brand. The vehicle models in the two data sets could be named differently in various information sources. For example, the vehicle brand “Mustang” in BearOil is named as “Yema,” a Chinese translation, in GUAZI. Therefore, if the automaker, make year (MY), and other technical specifications are the same, this study assumes that the records from different data sets describe an identical vehicle model.
• Fuzzily match vehicles within the same vehicle models. When no fuel economy information on BearOil is found to match the records listed in GUAZI, fuel economies of these vehicle records are assumed to be the median values of the fuel economies of vehicle trims within the same vehicle model. For example, the fuel economy for the vehicle record “Mazda Atenza 2018 2.5L Sports” in GUAZI cannot be found on BearOil. Therefore, the median fuel economy for the vehicle model “Mazda Atenza 2018” (33.6 MPG or 7.0 L/100 km) is assigned.
• Fuzzily match vehicle models by year. All records included in BearOil are vehicle models currently on the road, while all the records from GUAZI are for used cars, some of which are no longer sold in the new-car market. For example, vehicle records for the “Mazda Atenza” in MY 2012 to 2016 are still operating on the road in 2019 (that’s how we can collect these data records from GUAZI), but these models are not sold as new vehicles in 2019. As a result, vehicles produced in earlier years might not map their fuel economy information on BearOil. In this study, the fuel economy of old vehicle models is assumed to be the same as the fuel economy of the earliest vehicle models that can be queried from BearOil. For example, the fuel economy of records labeled as “Mazda Atenza” in MY 2012 to 2016 with a 2.5 L engine is considered to be 33.6 MPG (or 7.0 L/100 km), which is the fuel economy of “Mazda Atenza” in MY 2017.

#### Characterizing vehicle use intensity by class/size in the U.S.

For the U.S., only light-duty vehicles are considered in this study. As shown in Figure S2, based on the U.S. Environmental Protection Agency (EPA) classifications, which are primarily defined by the gross vehicle weight rating (fueleconomy.gov 2020), the records investigated in the U.S. are segmented into two-seaters, cars, station wagons, pickups, minivans (cargo vans are excluded), and SUVs. In the matched data set, 96,978 valid vehicle records with a vehicle age from 1 to 40 years are collected, where about 50% are cars (including 2% two-seaters, 45% cars, and 3% station wagons), 29% are SUVs, 17% are pickups, and 4% are minivans.

The relationships among vehicle class/size, the VMT and the labeled fuel economy in the U.S. are analyzed and compared. As shown in Figure S3A, larger passenger vehicles, including minivans, pickups, and SUVs, seem to travel more miles, on average, than smaller vehicles, such as two-seaters, cars, and station wagons. This observation is also verified with the t-test, which concludes the average per-vehicle annual VMT by minivans, pickups, and SUVs, are statistically significantly larger than those by cars (p value for one-tail statistic <0.0001). The t-test is valid for a large group even though it consists of non-normal distributed samples. Thus, the t-test analysis is adopted for comparing the records. This might be because these larger vehicles are more likely to be used for purposes other than just transporting people, and/or they are more frequently owned by people in rural areas in the U.S. (which might have longer commutes and fewer opportunities to use public transit) (U.S. Department of Transportation Federal Highway Administration, 2017). Meanwhile, smaller vehicles are owned more frequently by people in urbanized areas (U.S. Department of Transportation Federal Highway Administration, 2017) where there are relatively better public transit systems and higher ownership costs per mile of driving (such as higher fuel cost and labor cost). What’s more, the comparison of fuel economy (MPG) among different vehicle classes/sizes in Figure S3C clearly shows that the fuel economy of a larger vehicle tends to be statistically significantly lower than the fuel economy of a smaller vehicle (p value for one-tail statistic <0.0001). This might be because these larger vehicles are often heavier and/or equipped with more horsepower (U.S. EPA 2019). Thus, the households that own larger vehicles tend to consume more fuel than those that own smaller vehicles when they have the same per-vehicle annual VMT.

In addition, this correlation between the driving intensity and vehicle size seems to also exist within the vehicle class, as shown for the different car sizes in Figure S3B—the per-vehicle annual VMT seems positively correlated with the vehicle size. The comparisons are verified through t-tests for two sets of records. For example, the per-vehicle annual VMT of mini-compact cars is statistically significantly smaller than that of subcompact cars (p value for one-tail < 0.0001), and the per-vehicle annual VMT of subcompact cars is statistically significantly smaller than that of compact cars (p value for one-tail < 0.0001). Both the per-vehicle annual VMT of midsize cars and large cars are statistically significantly larger than that of compact cars. Meanwhile there is one exception, the per-vehicle annual VMT of midsize cars (including midsize cars and midsize station wagons) is statistically significantly higher than the per-vehicle annual VMT of large cars, including large cars only (p value for one-tail statistic <0.0001).

#### Characterizing vehicle use intensity by class/size in China

Passenger vehicles in China are classified into two major segments: (1) car or similar vehicles and (2) SUVs/crossovers/minivans, as shown in Figure S2. Each segment is further divided into three different groups by vehicle size: small, midsize, and large. As shown in Table S1, the group proportions in the dataset are close to the proportions of size segments in the Chinese passenger vehicle market by the end of 2018. The real-world data depicted in Table S1 was provided by the China Automotive Technology and Research Center (CATARC) (CATARC 2020). As shown in Table S1, small cars and small SUVs/crossovers/minivans are the most popular in both the new-vehicle and used-vehicle markets.

The relationships between the vehicle classification/size and per-vehicle annual VMT and vehicle-labeled fuel economy for the Chinese passenger vehicles are also analyzed and shown in Figures S3D–S3F). Figures S3D and S3E show a clear trend that a larger passenger vehicle is often driven more miles than a smaller passenger vehicle, regardless of whether the vehicle class segment is car or SUV/crossover/minivan. At the same time, Figure S3F clearly shows that a larger passenger vehicle has a lower fuel economy than that of a smaller passenger vehicle. The fuel economy (MPG) information shown in Figure S3F is converted from the labeled fuel consumption estimate (L/100 km) tested by MIIT. The vehicle fleet’s total fuel use is not only determined by the fuel economy of the vehicle but also depends on vehicle usage (usually in terms of per-vehicle annual VMT). Based on the above information, we can safely reach a conclusion that, unlike what is found in the U.S., a larger passenger vehicle could consume more fuel annually in China, and the fleetwide weighted-average fuel economy could be even lower if it is not only weighted by vehicle production or sales (such as in the U.S. CAFE) but also weighted by the actual per-vehicle annual VMT.

#### Data analysis on the vehicle records

In the U.S., the per-vehicle annual VMT for each vehicle record is calculated by referring to the BESTMILE (the best estimate of annual vehicle mileage by NHTS analysts)(NHTS 2017). In addition, each vehicle record is assigned a coefficient − *Final HH weight* in the NHTS for obtaining the weighting factor. The NHTS uses the variable − *Final HH weight* − to quantify the probability that the NHTS vehicle/household records can represent the real world. The per-vehicle annual VMT by class/size - presented in this study is a weighted average of the VMT for the vehicle records, as shown in Equation (S1).

(Equation S1) $$\bar{AVMT}=\sum_{i=1}^{n}AVMT_{i}\cdot k_{i}$$

Where, $\bar{AVMT}$ is the weighted average of the VMT for all vehicle records in the specific class/size, n is the number of vehicle records, $AVMT_{i}$ is the per-vehicle annual VMT (BESTMILE) for vehicle record i, and $k_{i}$ is the weighting factor of vehicle records.

The probability distributions of fuel economy and per-vehicle annual VMT in the U.S. are presented in Figure S4. Generally, the fuel economy of SUVs/pickups/minivans is statistically significantly (α = 1%) lower than the fuel economy of cars, and the per-vehicle annual VMT of SUVs/pickups/minivans is statistically significantly (α = 1%) larger than that of cars. Accordingly, the difference in annual fuel consumption between these two classes of vehicles is more statistically significant (the weighted average annual fuel consumption is 468 gallons for cars and 606 gallons for SUVs/pickups/minivans). In addition, 80% of cars annually consume about 96–880 gallons of fuel per vehicle, and 80% of the SUVs/pickups/minivans annually consume about 113–1,123 gallons of fuel per vehicle. The variable “final household weight” for vehicle records should be multiplied by the household share so that the results can properly account for the representations of these vehicle records in each state in the U.S. The total households in each state and their shares of the U.S. are obtained from the U.S. Census Bureau (U.S. Census Bureau 2020).

For China’s market, the collected data includes the information on cumulative VMT for each vehicle record. To obtain the representative per-vehicle annual VMT for each class/size, this study adopts the software @RISK® to fit (Monte Carlo method) the distribution, CVMT=δ(t,λ), of the cumulative VMTs by age (in month) of all vehicles in this class/size. CVMT is the cumulative VMT distribution, λ stands for the probability distribution parameters, t is the vehicle age. Accordingly, the per-vehicle annual VMT of this class/size can be obtained by Equation (S2). The per-vehicle annual VMT for Chinese vehicle records is transferred to log-transform in this study. The log-transform can decrease the variability of data, make data conform more closely to the normal distribution, and can be used to address data distributions less skewed (Feng et al., 2014).

(Equation S2) $$AVMT\left(t\right)=\frac{\partial \delta \left(t,\lambda \right)}{\partial t}$$

Where, AVMT(t) is the per-vehicle annual VMT at vehicle age t.

In addition, we obtain the vehicle stock by age information in China from the China Automotive Technology and Research Center. This study uses the software @RISK® to fit the distribution, t=φ(β), of the vehicle ages in the current Chinese market, provided by the CATARC (CATARC 2020). This study assumes that vehicle market shares by age (in years) are roughly unchanged by vehicle class. φ(β) is the age distribution in the market. β stands for the probability distribution parameters. Therefore, combined with the information on vehicle market shares by vehicle age (in years), the age-weighted average per-vehicle annual VMT by class/size can be calculated through Equation (S3).

(Equation S3) $$\bar{AVMT}=\underset{\text{Θ}}{\int }AVMT\left(t\right)\varphi \left(\beta \right)dt$$

Where, $\bar{AVMT}$ is the age-weighted average per-vehicle annual VMT (×1,000 mile) for a specific vehicle size/class,  Θ is the age range in the market. More details of the vehicle VMT calculation can be referred to Ou et al. (2020).

Figure S5 shows the per-vehicle annual VMT by vehicle size and class, along with the probability distributions of per-vehicle annual VMT in China. The probability distribution of per-vehicle annual VMT is right-skewed. For example, the average per-vehicle annual VMT of small cars is about 6,871 miles; and the confidence interval (±σ) with 68.3% probability of the per-vehicle annual VMT ranges from 5,669 miles to 8,345 miles.

The per-vehicle fuel use is obtained through Equation (S4).

(Equation S4) $$F_{m}={\bar{AVMT}}_{m}\cdot f_{m}$$

Where, ${\bar{AVMT}}_{m}$ is the probability distribution of average per-vehicle annual VMT (×1,000 miles) in vehicle class m. $f_{m}$ is the probability distribution of fuel consumption (gallons/mile) for vehicle class m. The fuel use of the vehicle fleet should consider the heterogeneity of the per-vehicle annual VMT and fuel consumption by vehicle size and class. Based on the data provided by CATARC, Table S2 lists the annual passenger vehicle sales in 2018 and the total passenger vehicle stock in China as of the end of 2018. The statistics considered only passenger vehicles with gasoline-powered internal combustion engines. Thus, the values shown in Table S2 would be slightly different from other sources.

#### SAF to address large vehicle usage underrepresentation

As shown in Section discussions, large vehicles are driven more and last longer. This contradicts the equal- or independent-usage assumption of the current sales-weighted approach. Starting from the $CAFE_{usage}$ formula in Equation (1), we can divide the annual driving distance ($d_{i}$) and vehicle lifetime ($l_{i}$) by reference values, such as the average values E[$d_{i}$] and E[$l_{i}$], the counterpart values of any vehicle type i, or virtually any constant values. For simplicity and ease of interpretation and without loss of generality, we use a specific vehicle type (i=1) as the reference point and define $k_{i}$ as the SAF for vehicle type i relative to vehicle type 1, as in Equation (S5):

(Equation S5) $$k_{i}=\frac{l_{i}d_{i}}{l_{1}d_{1}}=SAF_{i}$$

Then, $CAFE_{usage}$ in Equation (1) can be expressed as in Equation (S6):

(Equation S6) $$CAFE_{usage}=\;\frac{\sum_{i\in I}\left(k_{i}n_{i}\right)}{\sum_{i\in I}\left(k_{i}n_{i}f_{i}\right)}$$

This formula for CAFE calculation is usage-weighted (i.e., reflects the real-world vehicle lifetime fuel consumption). The high fuel consumption rate and popularity of large vehicles are reflected by $f_{i}$ and $n_{i}$, which are already in the sales-weighted formula in Equation (2). The higher annual usage and longer vehicle lifetime of large vehicles are now captured by the SAF, $k_{i}$, which is easy to interpret and can be estimated based on historical values of annual distance traveled and vehicle lifetime. If a vehicle type or class i is associated with a high SAF $k_{i}$ due to high annual distance traveled, long vehicle lifetime, or both, each unit of sales of vehicle i should be counted as $k_{i}$ units in the CAFE calculation.

Using the data in the U.S. and China, we estimate the SAF by vehicle class based on Equation (S5), as shown in Table 3. Table 3 presents the SAFs calculated through two methods. The SAFs calculated by Method #1 are based on a simple division of the average lifetime VMT by that of small cars for the U.S. or for China in Table 3, based on Equation (S5). For each vehicle class/size, the SAF in Method #2 is the average value of randomized SAFs for individual vehicles. These individual SAFs are also calculated based on Equation (S5), but the annual distance and vehicle lifetime are sampled from the distributions of per-vehicle annual VMTs and expected lifetimes. Method #2 aims to quantify the SAFs by considering the uncertainties of the lifetime VMTs of different vehicle classes/sizes. The Monte Carlo simulation method is adopted to calculate the mean values of the SAF distributions and their 90% confidence intervals (CI). Table 3 also presents the expected lifetimes of different vehicle classes/sizes, which are calculated based on the vehicle survival rates in the U.S. and China (Zheng et al., 2019; Davis and Boundy 2021). The details on calculating SAFs are presented by the following section - Calculation of sales adjustment factors by vehicle class/size, Figure S6, and Tables S3 and S4.

#### Calculation of sales adjustment factors by vehicle class/size

This study adopts two different methods to obtain the values of sales adjustment factors (SAFs), which are shown in Table 3 in the main text. In the 1^st^ method, SAF is quickly calculated through Equation (S5) in the main text with the inputs of average per-vehicle annual VMTs and expected lifetimes for different vehicle classes/sizes. Meanwhile, this method could ignore the uncertainties, or the distributions of annual vehicle miles traveled (VMT) and expected lifetime of a vehicle class/size. Therefore, the Method #2 considers the deficiency of the Method #1 and calculates the mean value of SAFs statistically. Equation (S7) shows the calculation in the Method #2.

(Equation S7) $$d\left(k_{i}\right)=\frac{d\left(LVMT_{i}\right)}{\bar{LVMT_{b}}}$$

Where, $d\left(k_{i}\right)$ is the distribution of SAF for vehicle class/size i. $d\left(LVMT_{i}\right)$ is the distribution of lifetime VMT for vehicle class/size i. $\bar{LVMT_{b}}$ is the mean value of lifetime VMT distribution of the benchmark class/size. The small car is the benchmark for the US and China on calculating the SAFs, respectively.

Firstly, the distribution of vehicle expected lifetime by vehicle class/size is calculated based on the vehicle survival rates in the U.S. and China (Zheng et al., 2019; Davis and Boundy 2021). Combining the distributions of per-vehicle annual VMTs and the distributions of expected lifetimes for different vehicle classes/sizes, we can obtain the distributions of lifetime VMTs for different vehicle classes/sizes. This combination is conducted under Monte Carlo Simulations with @RISK® software. The results are obtained by iterating 50,000 times with 10 simulations. It shows that the mean value of the lifetime VMT distribution for a U.S. small car is about 156,749.03 miles, and the mean value of the lifetime VMT distribution for a Chinese small car is about 191,824.93 km (119,194.49 miles). Their lifetime VMT distributions of small cars are presented in Figure S6.

Secondly, we obtain the distributions - $d\left(k_{i}\right)$ of SAFs by using the Monte Carlo Simulations by iterating 50,000 times with 10 times of simulations in @RISK®. Table 3 in the main text shows the mean values and the 90% of confidence intervals of mean values of the SAF distributions. Tables S3 and S4 presents the statistical results of SAFs in the U.S. and China by their vehicle class/size.

#### Quantifying impacts of SAF on regulation effectiveness and equity

This section explains the calculations leading to the results of SAF impacts in Figure 5. The “LDV average fuel economy change” in Figure 5 (a and b, left axis), labeled as Δ(CAFE), is calculated via Equation (S8), with the same parameter definitions in Equations (1) and (2), and Equations (S5)–(S6).

(Equation S8) $$\Delta \left(CAFE\right)=CAFE_{usage}-CAFE_{sales}=\;\frac{\sum_{i\in I}\left(k_{i}n_{i}\right)}{\sum_{i\in I}\left(k_{i}n_{i}f_{i}\right)}-\frac{\sum_{i\in I}n_{i}}{\sum_{i\in I}\left(n_{i}f_{i}\right)}$$

The “total compliance value of the fuel economy change” in Figure 5 (a and b, right axis), labeled as $CV_{all}$, is calculated via Equation (S9) below. The unit compliance value $CV_{unit}$ is assumed as $5.5 per 0.1 MPG (lower bound; current penalty rate) and $14 per 0.1 MPG (upper bound; proposed under on-going rulemaking) for the U.S. For China, $CV_{unit}$ is assumed to be $5.8 per 0.1 MPG (lower bound) and $8.8 per 0.1 MPG (upper bound) according to compliance cost estimates for 2030 and 2025 fuel consumption regulation scenarios by Wang et al. (2019).

(Equation S9) $$CV_{all}=\Delta \left(CAFE\right)*CV_{unit}*\sum_{i\in I}n_{i}$$

The “equity impact on fuel economy” in Figure 5 (c and d, left axis) for a given vehicle model or class j, labeled as $EquityMPG_{j}$, is calculated via Equation (S10), where $LD_{avg}$ is the sales-weighted lifetime distance as calculated via Equation (S11). The term $\frac{l_{j}d_{j}}{LD_{avg}f_{j}}$ represents the equivalent biased fuel economy due to usage underrepresentation or overrepresentation. It is calculated by dividing the actual lifetime distance $l_{j}d_{j}$ by the biased lifetime fuel consumption $LD_{avg}f_{j}$ that is based on the implicitly assumed uniform lifetime distance $LD_{avg}$.

(Equation S10) $$EquityMPG_{j}=\frac{1}{f_{j}}-\frac{1}{f_{j}^{*}}=\frac{1}{f_{j}}-\frac{l_{j}d_{j}}{LD_{avg}f_{j}}$$

(Equation S11) $$LD_{avg}=\frac{\sum_{i\in I}\left(n_{i}l_{i}d_{i}\right)}{\sum_{i\in I}n_{i}}$$

The “equity impact on compliance value” in Figure 5 (c and d, right axis) for a given vehicle model or class j, labeled as $EquityCV_{j}$, is calculated via Equation (S12), where $CV_{unit}$ is defined and specified in the same way as in Equation (S12).

(Equation S12) $$EquityCV_{j}=EquityMPG_{j}*CV_{unit}$$

All data and calculations for the U.S. market are available on Mendeley Data (https://doi.org/10.17632/98mcnkssgf.1).

#### Quantifying the relationship between household incomes and per-vehicle annual fuel consumptions

The ownership and usage of vehicles by households are featured by different economic factors such as household incomes, employment status, and driving experiences (Clark et al., 2016; Machado-León et al., 2016). This study focuses more on the relationship between household incomes and per-vehicle annual fuel consumption, which is majorly determined by vehicle type (MPG) and per-vehicle annual VMT. Therefore, this study furtherly quantifies the relationships for household incomes with vehicle types, and household incomes with per-vehicle annual VMT. The Chi-squared test is adopted to assess independence (Cohen 1980). With the Chi-squared test statistic being 5841055 and p value < 0.0001, the household incomes and vehicle types are not considered independent. With the Chi-squared test statistic being 2547449 and p-value < 0.0001, the household incomes and per-vehicle annual VMT are not considered independent either. Figure S8 shows the association plot, which is a way to visualize the table of Pearson residuals of the contingency table for (a) household income and vehicle type, and (b) household income and per-vehicle annual VMT (Meyer et al., 2003).

After proving the mutual dependence for (a) household income and vehicle type, and (b) household income and per-vehicle annual VMT, this study adopts the Cramer’s V test to quantify the correlations. For household incomes and vehicle type, the Cramer’s V coefficient is 0.162, which is regarded as a strong correlation between these two variables (Akoglu 2018). It indicates that household incomes are highly correlated with vehicle types. For household incomes and per-vehicle annual VMT, the Cramer’s V coefficient is 0.110, which is regarded as a moderate correlation between these two variables (Akoglu 2018). It indicates that the household incomes are moderately related with the per-vehicle annual VMT.

At last, this study adopts the Spearman’s rank correlation to quantify the monotonic correlation between household incomes and per-vehicle annual fuel consumption. The Pearson’s correlation coefficient for household incomes and per-vehicle annual fuel consumption is 0.358 with p value < 0.0001, which is regarded as a moderate monotonic correlation between these two variables (Akoglu 2018). It indicates that the households with lower incomes are likely to consume less fuels whereas the households with high incomes are likely to consume more fuels annually.

## Data Availability

The data used for analysis discussions are all provided in supplementary information. The input data used for the U.S. vehicle market analysis are publicly available from the National Household Travel Survey, nhts.ornl.gov. The input data including used car sales information and fuel economy information that are used for the Chinese vehicle market analysis are scraped from the commercial websites www.guazi.com, www.xiaoxiongouhao.com, and www.autohome.com.cn. The input data for the Chinese vehicle market and the data and calculation for the SAF impact on the U.S. market are provided on Mendeley Data (https://doi.org/10.17632/98mcnkssgf.1). Any additional information required to reanalyze the data reported in this paper is available from the lead contact upon request.
