# Supplementary material for: Improving the effectiveness and equity of fuel economy regulations with sales adjustment factors
Source: iScience. 2022 Aug 10;25(9):104902. doi: 10.1016/j.isci.2022.104902 (PMC9424598; doi:10.1016/j.isci.2022.104902)
Supplement: Document S1. Figures S1–S8 and Tables S1–S4 [file mmc1.pdf]

iScience, Volume *25*

Supplemental information

Improving the effectiveness and equity of fuel economy regulations with sales adjustment factors

Shiqi Ou, Zhenhong Lin, Chieh (Ross) Wang, Stacy Davis, Shasha Jiang, Michael Hilliard, Ho-Ling Hwang, Xu Hao, and Rujie Yu

Supplementary Information: Document S1

Supplementary Figures

Figure S1. Data scraping and mapping of vehicle information records in the U.S. and Chinese markets. Related to STAR Methods

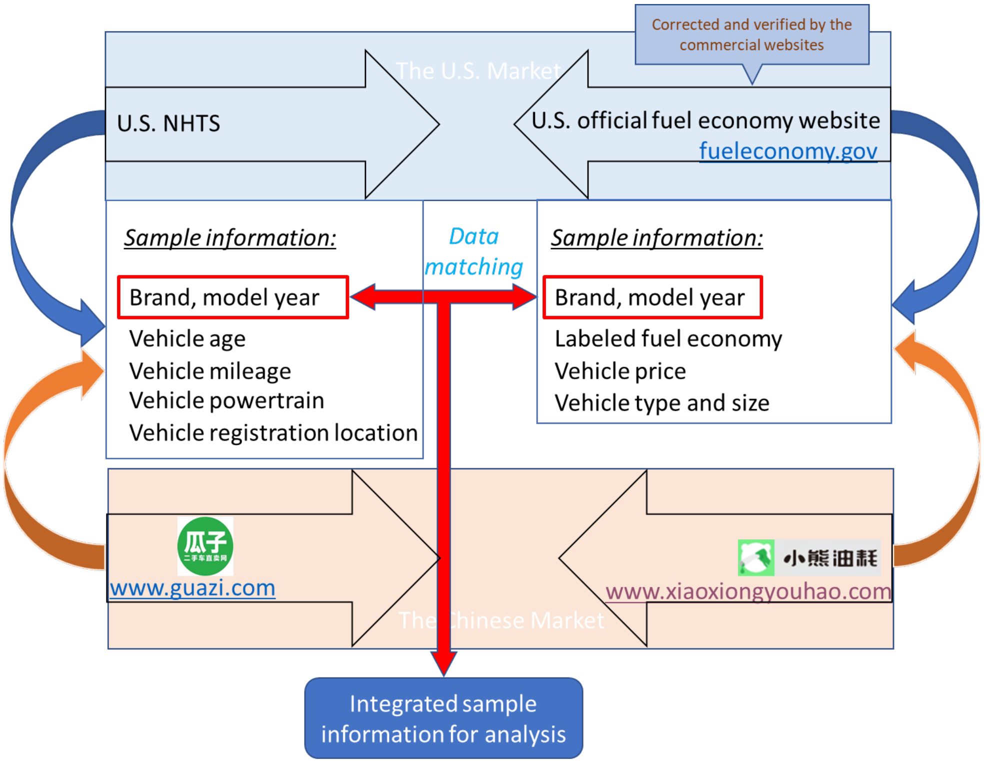

Figure S2. Classification of the vehicles. Related to STAR Methods

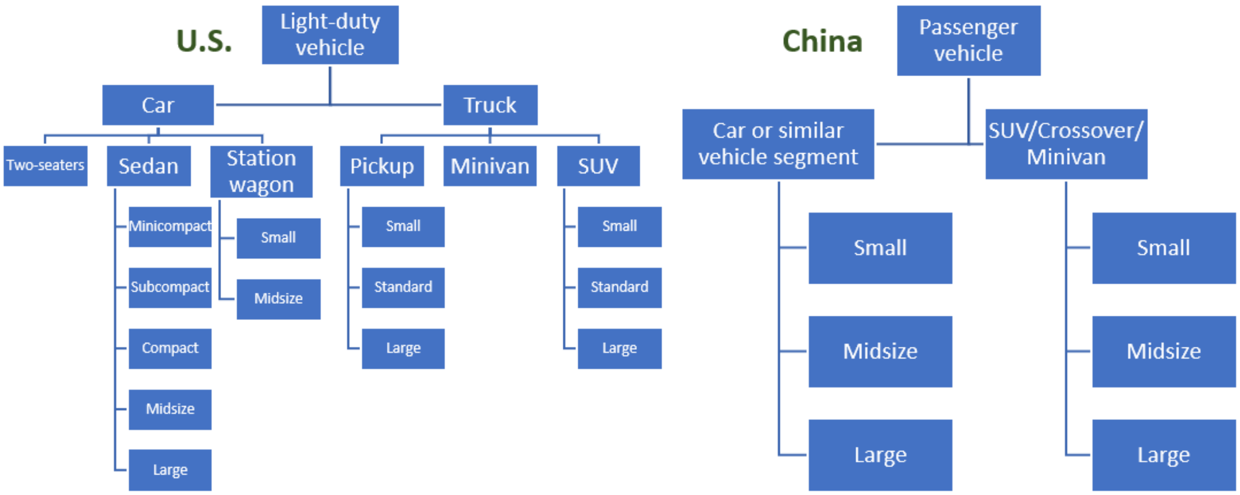

Figure S3. Heterogeneity of vehicle mileage by vehicle class and by vehicle age. Related to STAR Methods. The U.S. market: (a) light-duty vehicles, (b) cars, and (c) fuel economy range of the light-duty vehicle; China's market: (d) cars or similar vehicles, (e) SUVs/crossovers/minivans; and (f) the fuel economy range of privately-owned passenger vehicles in 2019. Error bars in (e) and (f) show the ranges of 1st quartile and 3rd quartile. Vehicle records for the U.S. market (a-c) are 96,978, obtained from the National Household Travel Survey (NHTS) (NHTS, 2017). Each vehicle record has been assigned a weighting value by the NHTS which represents the probability happening in the U.S. households. Vehicle records for vehicle miles travelled (VMT) calculation in China (d-e) are 169,292. Vehicle records for the fuel economy calculation in China (f) are 57,237. Details of data source are described in Supplementary Notes 1 and 2.

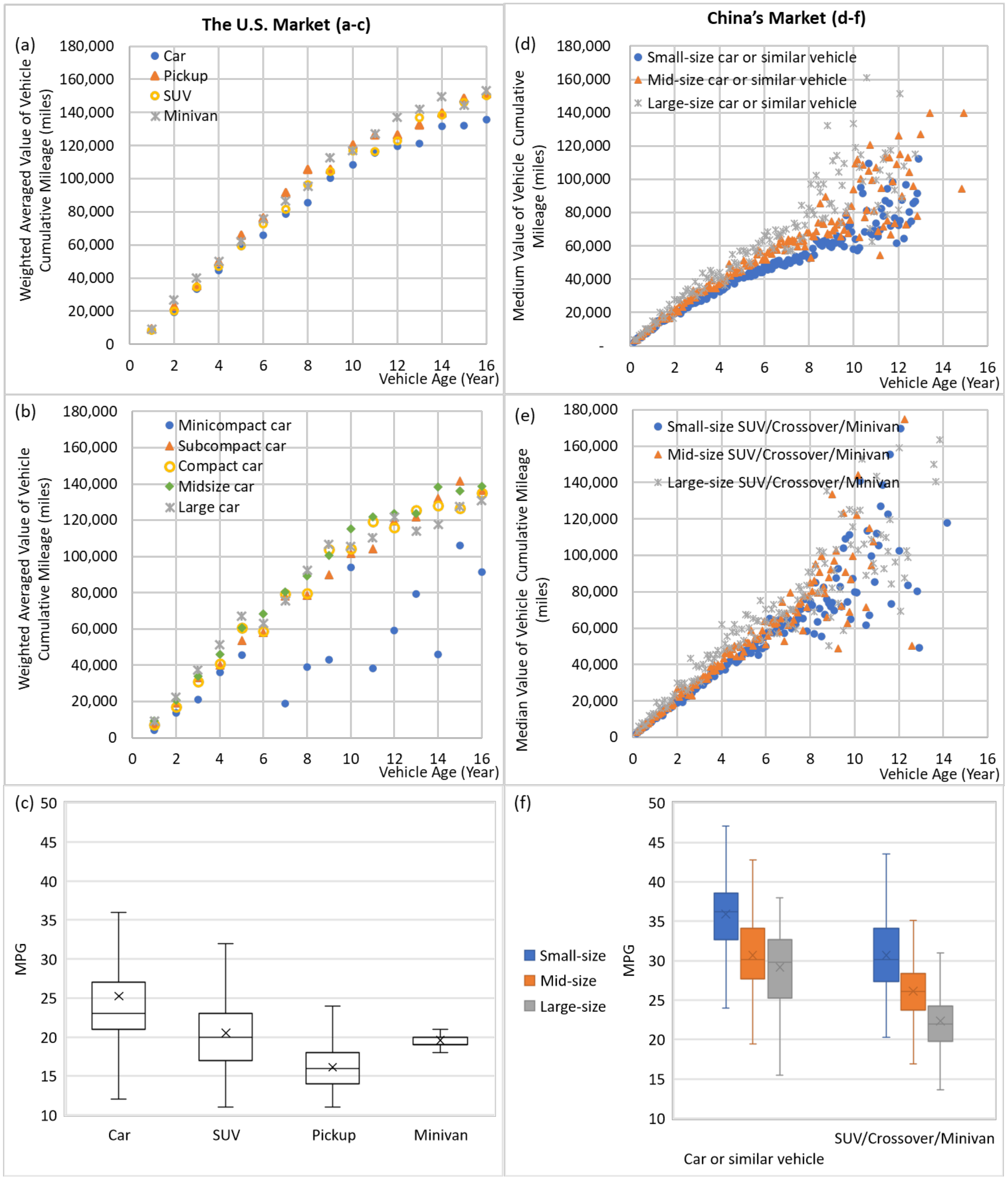

Figure S4. The probability density of fuel economy and per-vehicle annual VMT by vehicle class in the U.S.. Related to STAR Methods. The dashed line shows the moving average trend. Vehicle records for the VMT calculation are 96,978.

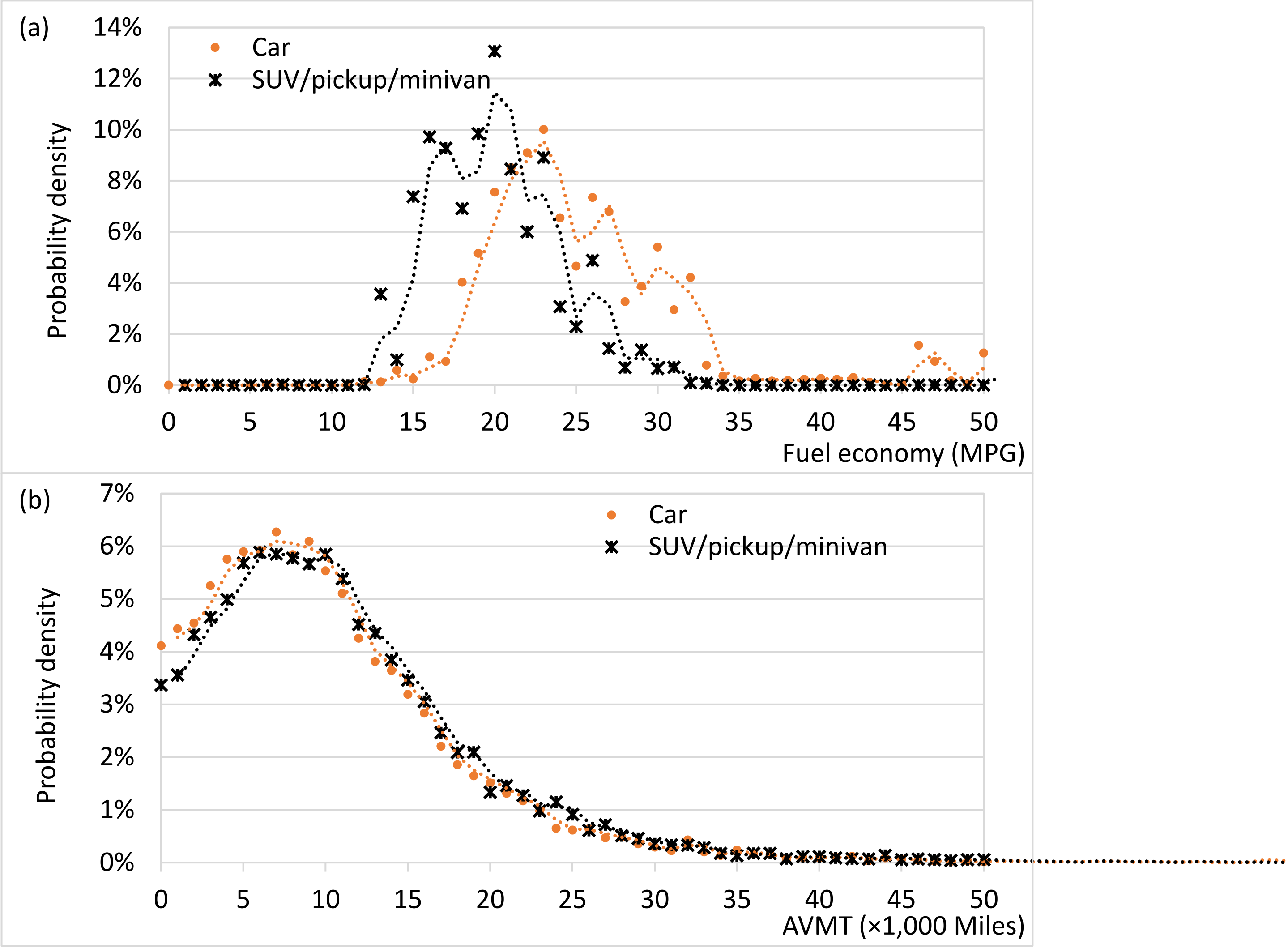

Figure S5. The fitting distributions of per-vehicle annual VMT by vehicle class/size for vehicles sold in China in 2004-2019. Related to STAR Methods. (a) Car or similar vehicle, and (b) SUV/crossover/minivan. The red lines and the numbers show the median values of the per-vehicle annual VMT in each vehicle class/size. Vehicle records for the VMT calculation are 169,292 collected in 2019. The fitting distributions are calculated based on the uncertainty quantification using @Risk®. The detailed methodology has been presented in Ou et al. 2019 (Ou et al., 2019).

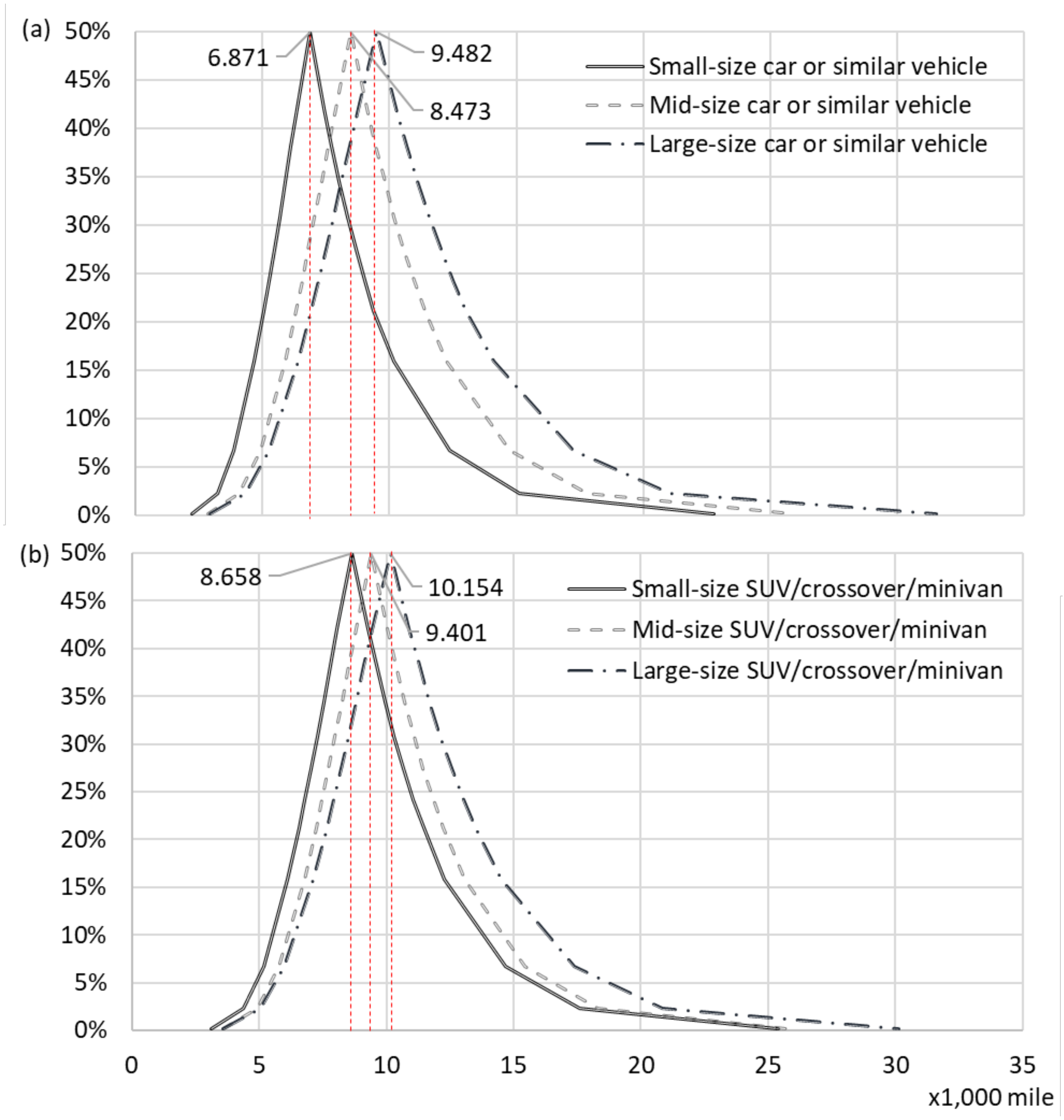

Figure S6. Distributions of lifetime vehicle miles travelled (VMT) for small cars and small cars in the U.S. and China, respectively. Related to STAR Methods. Vehicle records for calculating the lifetime VMT of small cars in the U.S. are 19,732. Vehicle records for calculating the lifetime VKT of small car in China's market are 23,047.

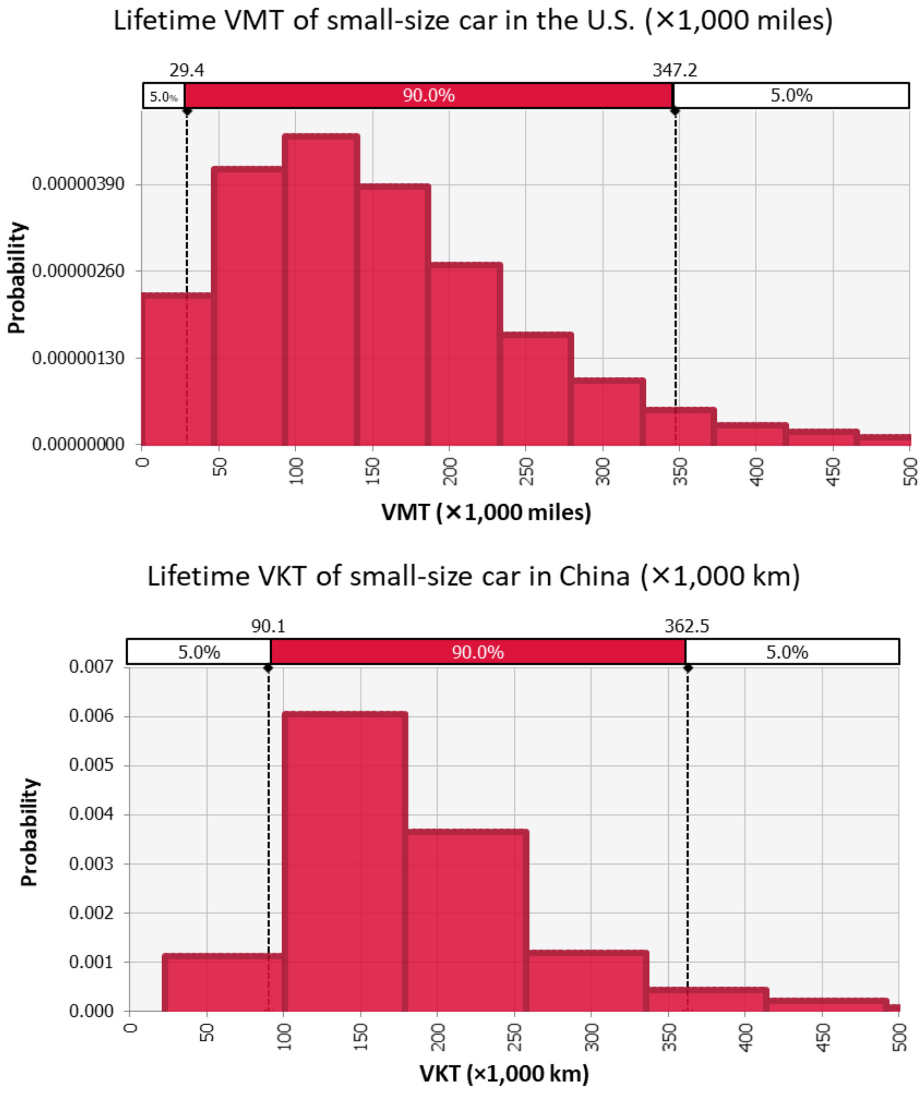

Figure S7. The Lorenz Curve of fuel consumption. Related to Results. All the U.S. households in the 2017 NHTS (Department of Transportation (DOT), 2017) are sorted by their annual fuel consumption from low to high. The further the curve is away from the dashed line; the less equal fuel consumption is among households. Certain households do consume more fuel than other households, whereas some households (approximately 11%) did not consume any fuel because they did not own a household vehicle. The graph is generated based on the information of a total of 129,696 households in the 2017 NHTS dataset (NHTS, 2017).

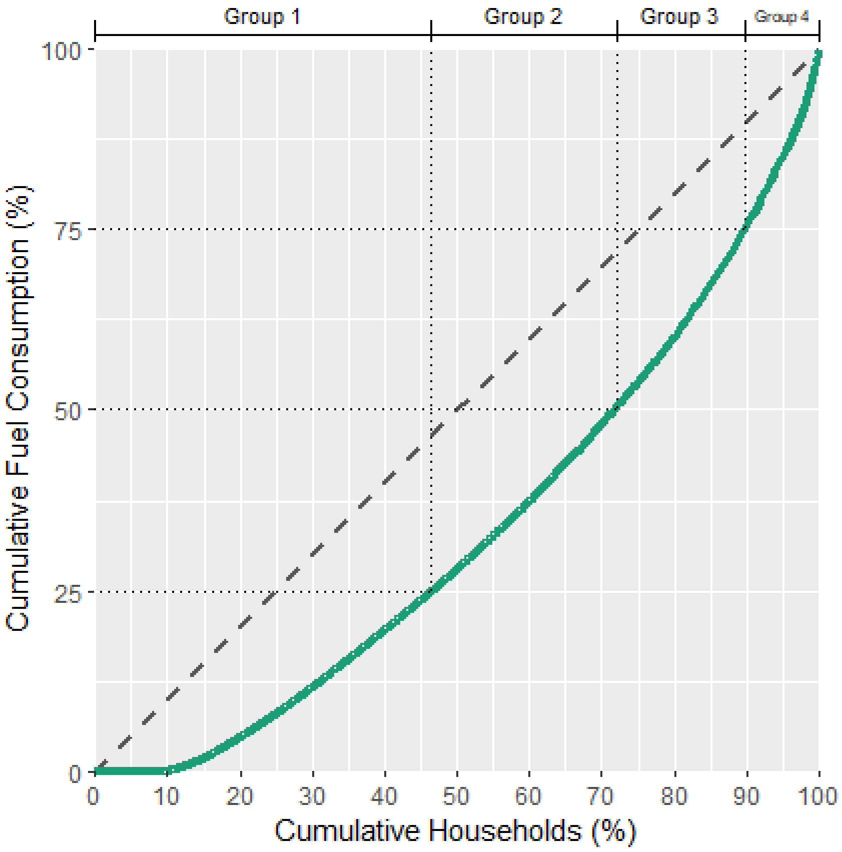

Figure S8. Association Plot of household income with vehicle types and per-vehicle annual VMT per vehicle in the U.S.. Related to Results. (a) The Pearson residuals between household income and vehicle type; (b) The Pearson residuals between household income and per-vehicle annual VMT. Each cell of this plot is represented by a rectangle with a signed height that is proportional to the corresponding Pearson residual and a width proportional to the square root of the expected counts. Blue means there are more observations in that cell than would be expected under the null hypothesis (independence). Red means there are fewer observations than would have been expected (Meyer et al., 2003). The original data is provided by NHTS (Department of Transportation (DOT), 2017).

(a)

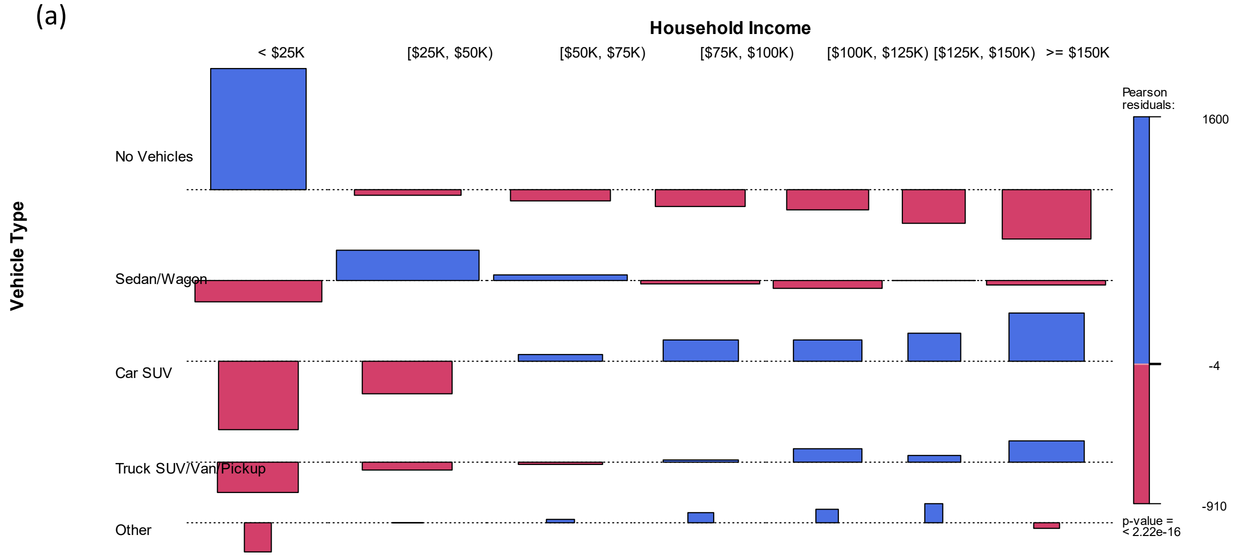

(b)

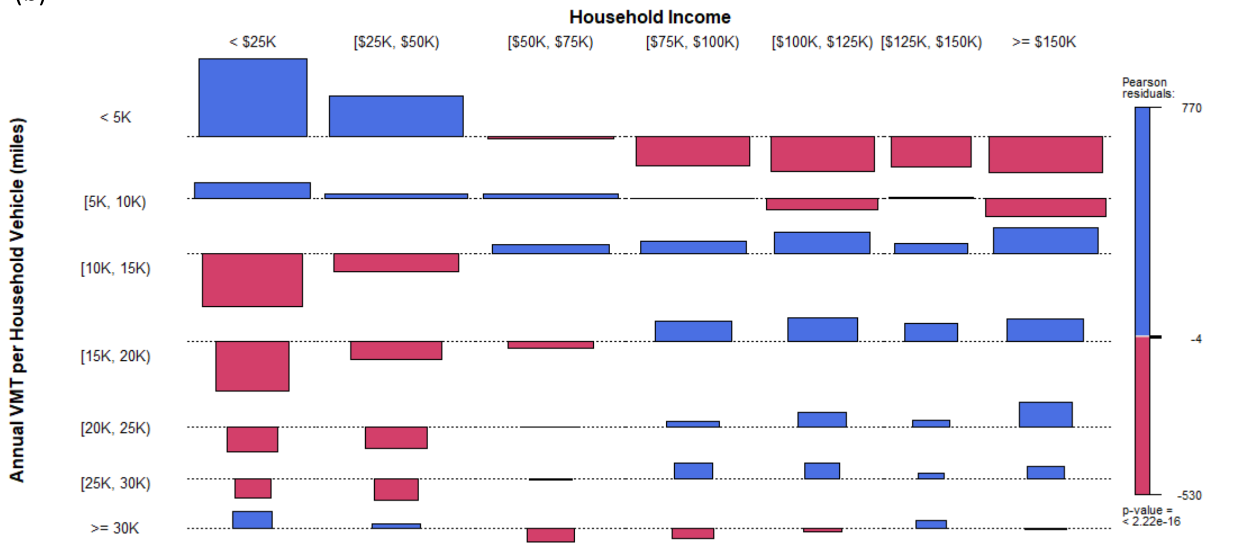

Supplementary Tables

Table S1. Data distribution and comparison with actual market population in China. Related to STAR Methods.

All passenger vehicle stock data is provided by the China Automotive Technology and Research Center (CATARC) (CATARC, 2020).

Class		Records for fuel economy-VMT	Proportion in record set	Proportion of passenger vehicle stock by class and size in the real-world market by the end of 2018
Car	Small	23,047	40.3%	52.2%
	Midsize	8,686	15.2%	12.0%
	Large	3,252	5.7%	3.2%
SUV/Crossover /Minivan	Small	14,116	24.7%	25.5%
	Midsize	5,509	9.6%	5.5%
	Large	2,627	4.6%	1.7%

Table S2. Passenger vehicle sales and stocks in China in 2018. Related to STAR Methods. Data is provided by the CATARC (CATARC, 2020).

**Class**		**Vehicle sales in 2018**	**Number of vehicles as of the end of 2018**
Car	Small	8,368,376	99,655,556
	Midsize	2,118,871	22,912,504
	Large	649,641	6,146,777
SUV/crossover/minivans	Small	9,554,154	48,672,943
	Midsize	2,108,723	10,432,707
	Large	369,417	3,259,759

Table S3. Statistical results of Sales Adjustment Factors of different passenger vehicle class/size in the U.S. Related to STAR Methods. Vehicle records for statistics are 96,978.

Statistical values	Car			SUV	Minivan	Pickup
	Small	Middle	Large			
**Minimum**	0.000529	0.00517	0.0021	0.00264	0.00607	0.00183
**Maximum**	14.506	32.19	71.66	17.641	401.32	55.28
**Mean**	1.001	1.146	1.243	1.283	1.88	1.319
**Mode**	0.618	0.604	0.59	0.797	0.8	0.623
**Median**	0.879	0.985	0.948	1.046	1.32	1.031
**Std Dev**	0.678	0.803	1.246	0.995	3.1	1.145
**Skewness**	1.9801	3.7576	9.0825	2.2529	52.5159	4.5923
**Kurtosis**	14.0967	65.88	283.28	14.06	5,881.64	114.77
**Probability distributions**						
**1.0%**	0.0691	0.12	0.122	0.0877	0.17	0.0861
**2.5%**	0.12	0.189	0.183	0.145	0.254	0.14
**5.0%**	0.188	0.267	0.252	0.218	0.345	0.205
**10.0%**	0.289	0.383	0.352	0.329	0.478	0.313
**20.0%**	0.456	0.555	0.51	0.514	0.688	0.493
**25.0%**	0.529	0.625	0.584	0.601	0.79	0.579
**30.0%**	0.599	0.697	0.652	0.688	0.888	0.666
**35.0%**	0.667	0.766	0.722	0.772	0.985	0.752
**40.0%**	0.736	0.838	0.795	0.854	1.09	0.841
**45.0%**	0.807	0.911	0.87	0.946	1.2	0.933
**50.0%**	0.879	0.985	0.948	1.046	1.32	1.031
**55.0%**	0.954	1.063	1.035	1.149	1.45	1.138
**60.0%**	1.031	1.144	1.129	1.264	1.59	1.256
**65.0%**	1.116	1.237	1.238	1.386	1.76	1.389
**70.0%**	1.207	1.338	1.367	1.531	1.95	1.539
**75.0%**	1.315	1.454	1.519	1.695	2.18	1.716
**80.0%**	1.445	1.604	1.702	1.885	2.49	1.93
**90.0%**	1.827	2.055	2.332	2.495	3.56	2.607
**95.0%**	2.213	2.534	3.093	3.122	4.94	3.379
**97.5%**	2.64	3.081	4.052	3.819	6.7	4.193
**99.0%**	3.26	3.892	5.831	4.806	10.1	5.481

Table S4. Statistical results of sales adjustment factors of different passenger vehicle class/size in China. Related to STAR Methods. Vehicle records for statistics are 57,237.

Statistical values	Car			SUV/Crossover/Minivan		
	Small	Middle	Large	Small	Middle	Large
**Minimum**	0.12	0.0788	0.133	0.0395	0.0642	0.0438
**Maximum**	18.843	22.084	36.675	24.448	17.497	19.04
**Mean**	1.000	1.196	1.383	1.341	1.424	1.579
**Mode**	0.752	0.93	1.111	0.959	1.229	1.209
**Median**	0.882	1.073	1.217	1.198	1.286	1.402
**Std Dev**	0.555	0.607	0.79	0.74	0.742	0.882
**Skewness**	5.242	4.8154	6.3553	3.5802	2.8843	3.2458
**Kurtosis**	79.5943	78.1286	140.9509	44.9199	26.1022	28.653
**Probability distributions**						
**1.0%**	0.339	0.423	0.468	0.330	0.360	0.387
**2.5%**	0.407	0.508	0.556	0.431	0.474	0.503
**5.0%**	0.469	0.580	0.642	0.531	0.579	0.620
**10.0%**	0.544	0.671	0.748	0.654	0.711	0.770
**20.0%**	0.645	0.792	0.886	0.822	0.891	0.963
**25.0%**	0.686	0.842	0.945	0.891	0.963	1.044
**30.0%**	0.725	0.888	1.000	0.953	1.028	1.118
**35.0%**	0.763	0.934	1.053	1.014	1.093	1.188
**40.0%**	0.802	0.979	1.106	1.074	1.157	1.257
**45.0%**	0.841	1.025	1.160	1.136	1.223	1.330
**50.0%**	0.882	1.073	1.217	1.198	1.286	1.402
**55.0%**	0.926	1.123	1.274	1.266	1.355	1.480
**60.0%**	0.974	1.178	1.340	1.337	1.432	1.566
**65.0%**	1.026	1.238	1.412	1.415	1.513	1.660
**70.0%**	1.085	1.308	1.497	1.504	1.601	1.768
**75.0%**	1.155	1.389	1.597	1.606	1.708	1.892
**80.0%**	1.242	1.487	1.720	1.731	1.836	2.043
**90.0%**	1.539	1.817	2.137	2.131	2.249	2.533
**95.0%**	1.890	2.185	2.624	2.596	2.695	3.058
**97.5%**	2.322	2.641	3.248	3.123	3.225	3.685
**99.0%**	3.027	3.351	4.235	3.983	4.065	4.728
